# A novel Kupffer cell-targeting nanoparticle system to Mitigate alcohol-associated liver disease

**DOI:** 10.1016/j.biomaterials.2025.123623

**Published:** 2025-08-09

**Authors:** Janitha M. Unagolla, Riley Flanagan, Kalindu Perera, Youbin Kim, Curtis Soloff, Emily Kaye, Angela Slitt, Jyothi U. Menon

**Affiliations:** a Department of Biomedical and Pharmaceutical Sciences, College of Pharmacy, University of Rhode Island, Kingston, RI, 02881, USA; b Department of Chemical Engineering, College of Engineering, University of Rhode Island, Kingston, RI, 02881, USA; c Department of Chemical Engineering, School of Engineering, Worcester Polytechnic Institute, Worcester, MA, 01609, USA; d Legorreta Cancer Center at Brown University, The Warren Alpert Medical School, Brown University, Providence, RI, 02912, USA; e Department of Biomedical Engineering, College of Engineering, Texas A&M University, College Station, TX, 77843, USA

**Keywords:** ALD, Kupffer cells, Nanoparticles, Biodistribution, Inflammation

## Abstract

Alcohol-associated liver disease (ALD) is a major cause of cirrhosis-related deaths worldwide. Due to its asymptomatic progression, early diagnosis and treatment are challenging, often resulting in severe complications. To address challenges associated with conventional treatments, including non-specificity and toxicity, we developed a poly lactic-co-glycolic acid nanoparticle (PLGA NP)-based drug delivery system that specifically targets Kupffer cells (KCs), which are major drivers of chronic liver disease progression. The PLGA NPs were loaded with the anti-inflammatory agent dexamethasone, and coated with carboxymethyl chitosan (CMC), a pH-responsive polymer that facilitates dexamethasone release in the acidic inflammatory environment. A semisynthetic bile acid, INT-777 was conjugated to the CMC-coated PLGA NPs to target G-protein coupled receptors expressed only on KCs in the liver. The NP formulation containing 1 % (w/v) CMC demonstrated significantly higher dexamethasone release at pH 6.0 and were cytocompatible. Cellular uptake studies with differentiated THP1 monocytes (modeling KCs) and HepG2 cells indicated a preferential uptake by THP1 cells, confirming INT-777 specificity. In an ALD mouse model developed through *ad libitum* ethanol feeding (5 % (v/v) for 10 days), biodistribution studies showed significant NP accumulation in the liver at 24 and 72h following administration. Treatment with CMC-coated PLGA NPs containing dexamethasone and INT-777 led to significantly decreased serum aspartate aminotransferase, alanine aminotransferase, and pro-inflammatory cytokines levels, and reduced liver inflammation as confirmed by Hematoxylin and Eosin, and Oil red O staining. These findings demonstrate that CMC-coated PLGA NPs hold significant potential as a treatment option for ALD-associated inflammation.

## 1. Introduction

Alcohol-associated liver disease (ALD) is a common chronic liver condition caused by excessive alcohol consumption, which is defined as consuming >1 standard drink per day for women and >2 standard drinks per day for men, where 1 standard drink contains about 14g of alcohol according to the United States standard [1–3]. Chronic liver inflammation resulting from alcohol abuse and other factors is a significant public health concern. The progression of ALD follows specific stages, starting with simple liver fat accumulation (steatosis), advancing to steatohepatitis (fat accumulation with inflammation), then to cirrhosis (advanced liver scarring), and in severe cases, hepatocellular carcinoma [4,5]. Around half of all liver cirrhosis deaths globally are linked to alcohol abuse. In the United States, alcohol has resulted in rising rates of mortality from cirrhosis, especially among the 25–34 age group [6]. It is estimated that from 2019 to 2040, about 1,003,400 people will die from chronic ALD in the United States, with 35 % of them under the age of 55 [7]. In 2016, ALD became the leading indication for liver transplant waitlist addition in the United States by surpassing the hepatitis C virus [8]. Prolonged alcohol consumption is also associated with increased cancer risk; ALD currently contributes to one-third of the world’s cases of primary Hepatocellular Carcinoma (HCC).

Most patients with early to mid-stage ALD, such as hepatic steatosis or mild steatohepatitis, are asymptomatic. As a result, a diagnosis of ALD is often not made until the later stages of the disease. For those individuals who are diagnosed, alcohol abstinence and proper nutrition are advised to prevent disease progression. Indeed, early stages of the disease, like steatosis, can be reversed if alcohol consumption is stopped [1]. There are limited therapeutic options available for patients with late-stage ALD. The main treatments include prednisolone -a corticosteroid, and pentoxifylline -a phosphodiesterase inhibitor used for patients in whom corticosteroids are contraindicated or ineffective. It is important to note that these medications only help to reduce short-term mortality [9,10]. A major challenge of current anti-inflammatory, anti-viral, and immunosuppressive therapies for liver inflammation is their inconsistent effectiveness and safety issues [11]. Furthermore, several therapies in clinical trials for hepatic inflammation have failed due to non-specificity and toxicity [12]. There is an urgent need for effective methods of delivering therapies to the inflamed liver to provide sustained treatment and prevent disease progression.

Therapies being developed currently target the pathogenesis of ALD, including inflammation (for example, anti-tumor necrosis factor (TNF) therapy, interleukin (IL)-22, glucocorticoids, steroids, β inhibitors, granulocyte-colony stimulating factor), oxidative stress (for example, S-adenosylmethionine, betaine, natural antioxidants), fibrosis (for example, transforming growth factor-β inhibitors, phosphodiesterase inhibitors, PPAR agonists), gut barrier dysfunction and microbial dysbiosis (for example, probiotics and antibiotics), and other processes [1,13]. While there has been heavy focus on hepatocytes which make up about 80 % of the liver volume, Kupffer cells (KCs) - the resident macrophages in the liver, have received relatively less attention despite playing a major role in initiating and promoting liver inflammation [14]. In liver disease, activated KCs produce transforming growth factor-beta (TGF-β) and platelet-derived growth factor, both of which are profibrogenic factors. These factors, combined with the generation of reactive oxygen species, inflammatory cytokines, and lipid peroxidation, activate hepatic stellate cells (HSCs). The activated HSCs in turn transdifferentiate into myofibroblasts, contributing to the development of liver fibrosis [15,16]. There has been significant interest recently to manipulate KC-mediated immune responses to counter liver inflammation [12,17]. The stimulation of G-protein-coupled bile acid receptor (Gpbar1), also known as TGR5, which is expressed exclusively by KCs but not hepatocytes in the liver, is of interest as it can promote an anti-inflammatory phenotype by suppressing pro-inflammatory cytokine release via cyclic AMP (cAMP) signaling [18,19].

In this research, we report the development of a novel ligand-decorated NP formulation that can target and stimulate the Gpbar1 expressed in KCs to have a potent anti-inflammatory effect. The formulation consisted of an FDA-approved poly lactic-co-glycolic acid (PLGA) polymer core, which is used to encapsulate the anti-inflammatory drug dexamethasone (DEX) for delivery to the liver. The DEX encapsulated PLGA NPs were coated with carboxymethyl chitosan (CMC), a pH-responsive biocompatible polymer, allowing for the triggered release of DEX in response to the acidic environment in the inflamed liver. CMC is amphoteric in nature as it contains both −COOH groups and −NH_2_ groups. At highly acidic pH (<2), the protonation of the amine groups dominate while at highly basic pH, the deprotonation of carboxyl groups is seen. However, as the pH increases from acidic to neutral conditions, the interactions between the partially ionized COOH and NH2 groups can lead to crosslinks within the polymer chain leading to shrinkage and release of the encapsulated drug. [20–22]. Finally, the NPs were surface decorated with INT-777, a highly selective and potent semisynthetic bile acid agonist of Gpbar1 that can attenuate pro-inflammatory cytokine production by KCs. The objective of this research was to develop a dual-functional NP-based drug delivery platform surface decorated with INT-777 for simultaneous targeting and activation of Gpbar1 for ALD treatment. Detailed characterization of the NP formulation as well as *in vitro* and *in vivo* investigation of its targeting and therapeutic efficacy was carried out. The *in vivo* investigation of NP biodistribution and therapeutic efficacy was carried out using the National Institute on Alcohol Abuse and Alcoholism (NIAAA) Bin Gao model of chronic ALD with the goal of precisely targeting KCs in inflamed livers for drug delivery to prevent the progression of chronic ALD.

## 2. Methods

### 2.1. Synthesis of pH responsive PC NPs

Three different batches of PLGA NPs were prepared by varying the CMC (AK Scientific, Union City, CA) concentration, using standard single emulsion solvent evaporation technique. Briefly, amine-modified PLGA (Nanosoft Polymers, Winston-Salem, NC) was dissolved in dichloromethane (DCM, Fisher Scientific, Hampton, NH) and methanol (Fisher Scientific) 9:1 ratio mixture and added to the 5 % (w/v) poly vinyl alcohol (PVA, Sigma-Aldrich, St. Louis, MO) solution dropwise. This emulsion was then sonicated at 60 W for 3 min (30s on, 15s off) using a probe sonicator (Fisher Scientific, PA) following which it was allowed to stir overnight at 500 rpm to evaporate the DCM and methanol. The PLGA NPs were then collected and washed with deionized water by ultracentrifugation (Optima L-100XP, Beckman Coulter, USA) at 35,000 g for 35 min at 4°C. After this step, the NP suspension was added dropwise to 1 % (w/v) PVA solution containing different concentrations of CMC - 0.2 % (w/v), 0.5 % (w/v), and 1 % (w/v), and kept for 6 h under continuous stirring at 500 rpm to allow physical coating of CMC on the PLGA NPs. Our preliminary studies confirmed that CMC concentrations above 1 % (w/v) resulted in large particles (>500 nm), which were unstable in ultrapure water. Therefore, concentrations below 1 % w/v were used for NP optimization. The samples were centrifuged twice, and NPs were collected after freeze-drying (Labconco, USA). The NPs with three different CMC concentrations will be hereafter referred to as PC-0.2, PC-0.5, and PC-1.

For the DEX-loaded NP (PC + DEX) preparation, DEX (Alfa Aesar, Harverhill, MA) was dissolved in methanol, and added to the PLGA-DCM solution at a 1:10 drug to polymer ratio. The resulting solution was added dropwise to the 5 % (w/v) PVA solution followed by the steps described above.

For coumarin-6 or Nile red fluorescent dye-loaded NP preparation, the dye was dissolved in methanol and PLGA was dissolved in DCM with 0.1:10 dye to polymer ratio, followed by the steps described above.

### 2.2. Preparation of INT-777 conjugated NPs

NPs were conjugated with INT-777 (MedChemExpress, Monmouth Junction, NJ) using carbodiimide chemistry. Briefly, INT-777 (0.5 mg/ml) was added to MES buffer (pH 6.0) followed by addition of 40 mg of N-(3-Dimethylaminopropyl)-N′-ethylcarbodiimide (EDC) (ThermoFisher Scientific, Waltham, MA) and 40 mg of N-Hydroxysuccinimide (NHS) (Fisher Scientific, Waltham, MA). After 30 min, 5 mg of PC NPs were added to the solution and kept overnight in shaker at room temperature. The NP suspension was centrifuge at 35,000 rpm for 30 min at 4 °C and pellet containing NPs were freeze-dried.

### 2.3. Characterization of size and morphology of the NPs

Size distribution, polydispersity index (PDI), and zeta potential (ZP) of the NPs were determined by dynamic light scattering (DLS, NanoZS, Malvern, USA). Samples (n = 3) for size and ZP were prepared by dispensing NPs in 1–1.5 ml of ultrapure water and subjecting the suspension to a DLS count rate of 200–300 kcps [23]. The shape and surface morphology of the NPs pre and post CMC coating were analyzed by using the secondary electron detector of Sigma VP field emission scanning electron microscope (SEM) at an electron accelerating voltage of 5 kV. Samples for SEM were prepared by drying a diluted drop of NP suspension (0.2 mg/ml) in ultrapure water on a silicon wafer secured on a SEM sub using carbon tape, followed by gold sputter coating. The CMC coating and shape of the NPs were further analyzed by using a JEM-2100 (JOEL, Japan) transmission electron microscope (TEM); samples were prepared by placing a drop of NP suspension (1 mg/ml) on a copper TEM grid, and excess liquid was drawn off using a filter paper, followed by negative staining using UranyLess 22409 and three dips in ultrapure water, after which the samples were left to air-dry overnight [24]. The CMC coating layer on NPs was confirmed using attenuated total reflectance Fourier transform infrared spectroscopy (ATR FT-IR) spectra of NPs recorded in the 400–4000 cm^−1^ range at a 2 cm^−1^ spectral resolution and 50 scans per sample using an IRTracer-100 spectrometer (Shimadzu, Japan).

### 2.4. NP stability study

To evaluate the *in vitro* stability of NPs, PC-0.2, PC-0.5, and PC-1, were suspended separately in ultrapure deionized water, 1X phosphate buffered saline (PBS) (Sigma Aldrich, St Louis, MO), and 10 % fetal bovine serum (FBS) (Innovative Bioscience, Benicia, CA) at pH 6.0 and pH 7.4. The samples were kept at 37°C for 5 days and particle sizes were assessed every 24 h. The study was carried out in triplicates to measure particle diameters, PDI and ZP. Particle diameters at each timepoint were compared to the diameters at the initial timepoint to assess variations in diameters over time.

### 2.5. In vitro drug release study

To assess the release profile of DEX from the NPs at different pH, 2 mg of DEX-loaded PC NPs were dispersed in 2 ml of PBS and transferred to a dialysis bag (molecular weight cut-off (MWCO) of 12–14 kDa-SpectrumPor, Repligen, Waltham, MA) immersed in 5 ml of PBS in a screw-capped 50 ml tube and placed on an orbital sharker which was set at 60 rpm, maintaining 37.4 ± 0.5 °C for 21 days. To check the pH-responsive release of DEX, release study was conducted on pH 6.0 and pH 7.4 PBS solutions. At predetermined time intervals, 1 ml of dialysate was collected and replaced with same volume of PBS at the same pH. The released DEX at each time interval was determined using UV spectrophotometer at 243 nm.

The encapsulation efficiency of the DEX was determined using direct encapsulation method. Briefly, 2 mg of PC + DEX NPs were dissolved in acetonitrile and the resultant solution was then centrifuged at 15,000 g for 15 min to separate any undissolved parts. Then the supernatant was collected and measured absorbance at 243 nm using UV spectrophotometer. The encapsulation efficiency (EE) and loading degree (LD) were calculated as follows:

$$EE(\%)=\frac{\text{Amount}\;\text{of}\;\text{drug}\;\text{in}\;\text{NPs}}{\text{Initial}\;\text{amount}\;\text{of}\;\text{drug}}\times 100$$

$$LD=\frac{\text{Amount}\;\text{of}\;\text{drug}\;\text{in}\;\text{NPs}}{\text{Total}\;\text{weight}\;\text{of}\;\text{NP}+\text{drug}}$$

Based on the results of the stability and drug release studies, PC-1 NPs were chosen for further assessment *in vitro* and *in vivo*.

### 2.6. Cell culture

Human hepatocellular carcinoma cell line HepG2 cells were obtained from American Type Culture Collection (ATCC, Manassas, VA, USA). Cells were cultured in Dulbecco’s modified Eagle’s medium (DMEM) (GenClone, Genesee Scientific, El Cajon, CA, USA) containing 10 % FBS and 1 % penicillin-streptomycin (PS) (Gibco, Thermo Fisher, Waltham, MA, USA) at 37°C in a 5 % CO_2_ incubator. The cells were subcultured at 80–90 % confluency using 0.25 % trypsin EDTA (Gibco, Thermo Fisher, Waltham, MA, USA). The HepG2 cells were used as a model hepatocyte cell line for *in vitro* studies. Human monocytic cell line THP1 cells were cultured in Rosewell Park Memorial Institute (RPMI) 1640 medium (Genesee Scientific, El Cajon, CA, USA) containing 10 % FBS and 1 % PS at 37 °C in a 5 % CO_2_ incubator. Monocytes were differentiated into macrophages (DTHP1) in the presence of 50 nM of phorbol-12-myristate 13-acetate (PMA) (PeproTech, Cranbury, NJ) for 24 h. After complete differentiation, DTHP1 macrophages were subjected to trypsinization as described above and were directly used for the experiments. DTHP1 cells were used to model KCs for the *in vitro* experiments.

### 2.7. In vitro cytocompatibility studies

NPs cytocompatibility against HepG2 and DTHP1 cells were evaluated using water-soluble tetrazolium 1 (WST-8) (MedChemExpress, Monmouth Junction, NJ, USA) assays. HepG2 and DTHP1 cells were seeded separately in 96 well plates at 20,000 cells/well, respectively and incubated for 24 h to facilitate cell attachment. The cells were then treated with varying concentrations (0,100, 250, 500, 1000, 2000 μg/ml) of PC-1 NPs and INT-777-tagged PC-1 NPs (PC-1+INT) suspended in media and incubated for 24 h at 37 °C (n = 4 per concentration). After 24 h, the media with NPs were removed and gently washed with PBS, following which WST-8 assay was carried out according to the manufacturer’s instructions. After 2 h of incubation with WST-8 reagent, absorbance readings were collected at 450 nm using Synergy H1 microplate reader (Biotek, Winooski, VT, USA) and percentage cell viability was calculated related to the control group.

### 2.8. Cell uptake studies

To determine targeting efficacy and uptake *in vitro*, coumarin-6 loaded PC-1 NPs with and without INT-777 were used. The HepG2 and DTHP1 cells were seeded in a 96 well plate at 20,000 cells/well, respectively. After 24 h of incubation, cells were treated with 1 μg/ml lipopolysaccharide (LPS) (Invitrogen, Carlsbad, CA, USA) and 0.16 % (v/v) ethanol (EtOH) (200 Proof, Decon Labs, King of Prussia, PA, USA) for 24 h to mimic ALD conditions [25]. Cells not treated with LPS and EtOH were used as controls. After 24 h of LPS+0.16 % EtOH treatment, these cells were subjected to FBS starvation for 2 h and followed by treatment with varying concentrations (0, 250, 500, 750, 1000 μg/ml) of NP formulations. After 2 h, the cells were lysed with 1 % Triton X-100 and the uptake of NPs was quantified by measuring fluorescence intensity (excitation- 457 nm and emission- 501 nm) and normalizing the particle concentration with total protein content in each well, determined by Pierce BCA protein assay. For qualitative analysis, uptake was visualized using an EVOS FL Auto Imaging system (ThermoFisher Scientific, Waltham, MA, USA). The uptake study followed the same procedure as described above until NP treatment. After 2 h NP treatment, the cells were washed with PBS and then fixed using 4 % paraformaldehyde (PFA) and nuclei stained with DAPI prior to imaging. Fiji (ImageJ flavors) software [26] was used to quantify the NP uptake as an additional quantitative measurement. The images were taken at same fluorescence intensity, and the same threshold was used during the Fiji analysis to ensure consistency.

### 2.9. In vitro therapeutic efficacy studies

To investigate the therapeutic efficacy of the NP system, levels of pro-inflammatory cytokines, such as Interleukin-1beta (IL-1β), IL-6, and tumor necrosis factor-alpha (TNF-α), were evaluated using IL-1β (BioLegend, 437004, San Diego, CA, USA), IL-6 (BioLegend, 430504), and TNF-α (BioLegend, 430204) enzyme-linked immunosorbent assay (ELISA) kits. The expected minimum detectable concentrations for these ELISA kits are 0.5 pg/ml, 4 pg/ml, and 2 pg/ml, respectively. Since macrophages are primarily responsible for inflammatory cytokine production in ALD, only DTHP1 cells were used for this study. The DTHP1 cells were seeded into a 96-well plate at 50,000 cells/well. After 24 h of incubation, cells were concurrently treated with 1 μg/ml LPS+0.16 % EtOH and the free drugs or NPs. Briefly, control, ALD control (1 μg/ml LPS+0.16 % EtOH), Free INT (0.1 μM and 1 μM), Free DEX (0.2 μM and 2 μM), PC-1 NPs, PC-1+INT NPs, PC-1+DEX NPs, and PC-1+DEX + INT NPs were used in this study. The NPs were tested at two different concentrations: 500 μg/ml and 1000 μg/ml. Therefore, the concentrations of free INT and free DEX were chosen to match the amounts encapsulated within the NPs at the 500 and 1000 μg/ml concentrations. After 6 h and 24 h of concurrent treatment, IL-1β, IL-6, and TNF-α cytokine levels were analyzed according to the manufacturer’s protocol.

### 2.10. Cyclic adenosine monophosphate (cAMP) assay

Chronic alcohol exposure has been associated with decreased cAMP levels in liver macrophages. Therefore, the cAMP activity of the DTHP1 cells was analyzed using cAMP assay kit (Abcam, Boston, MA, USA) according to the manufacturer’s instructions. Briefly, DTHP1 cells were cultured in white 96 well plates for 24 h at a seeding density of 50,000 cells/well. After 24 h of incubation, the same treatment groups as above, i.e., control, ALD control (1 μg/ml LPS+0.16 % EtOH), Free INT (0.1 μM and 1 μM), Free DEX (0.2 μM and 2 μM), PC-1 NPs, PC-1+INT NPs, PC-1+DEX NPs, and PC-1+DEX + INT NPs were used. The cAMP assay was carried out after the predetermined treatment incubations of 6 h and 24 h, according to the manufacturer’s protocol. Control cells without LPS + EtOH treatments and ALD controls without PC-1 NPs were used to measure the relative cAMP activity of the treatment groups. After the predetermined time points, the media was removed, and cells were lysed using cell lysis buffer and conducted the cAMP ELISA according to the manufacturer’s protocol. The absorbance was measured using a Synergy H1 microplate reader (Biotek, Winooski, VT, USA).

### 2.11. In vivo studies

#### 2.11.1. Developing ALD in a mouse model

An NIAAA model was used to evaluate the efficacy of the NP system *in vivo*. Nine weeks old C57BL/6 N female mice were purchased from Jackson Laboratory (ME, USA). All the animal studies were carried out according to the approved Institutional Animal Care and Use Committee (IACUC) protocol of University of Rhode Island. After the initial acclimatization period, mice weights were recorded and a second acclimatization was initiated using the control Lieber-DeCarli liquid diet (Bio-Serv, product no. F1259SP) for 5 days. Following this acclimatization, mice were divided into two groups: the Liber-Decarli control liquid diet group and Lieber-DeCarli EtOH diet group. The EtOH group was gradually introduced to the EtOH liquid diet (Bio-Serv, product no. F1258SP) starting with 1 % (v/v) EtOH and maltose dextrin. A direct switch from the control diet to the Lieber-DeCali EtOH diet containing 5 % (v/v) EtOH led to decreased food consumption and significant weight loss in the mice for several days. Therefore, we implemented a graduate increase in the EtOH concentration, starting at 1 % (v/v) EtOH and increasing by 1 % every day until the final concentration of 5 % (v/v) EtOH was reached. Due to the gradual increase of EtOH, consumption of the Lieber-DeCarli diet and the mouse weights did not drastically reduce. The daily consumption of the liquid diet and body weight of each mouse were both recorded. Mice were fed EtOH diet for 10 days to develop ALD, following which a biodistribution study and NP treatment efficacy study was conducted on the ALD mouse models.

#### 2.11.2. Biodistribution study

On day 11 of the EtOH diet, mice were given coumarin-6 or Nile red dye-containing NPs (PC-1 NPs and PC-1+INT NPs). Both control diet-fed and 5 %EtOH diet-fed mice were treated with 200 μl of 2 mg/ml NP suspension in sterile PBS solution intraperitonially. After 24 h and 72 h mice were deep anesthetized using isoflurane and subjected to cardiac puncture and finally euthanized by cervical dislocation. The blood was collected, and resultant plasma was used to determine the aspartate aminotransferase (AST) and alanine transaminase (ALT). After euthanization, organs such as liver, spleen, kidney, heart, and lung were harvested and fixed in 4 % PFA. After overnight fixation in 4 % PFA at 4°C, organs were washed in PBS and put in a 30 % sucrose solution and after 2 days, organs were fixed in optimal cutting temperature (OCT) solution. OCT embedded tissues were sectioned to 10 μm thick sections using cryostat (Leica CM3050S, Deer Park, IL, USA). The tissue slides were washed in PBS and running tap water and finally covered with coverslip using water-based mounting media (VECTASHIELD antifade mounting medium, Cole-Parmer, IL, USA). The slides were observed using a confocal fluorescence microscopy (Nikon Ti2, NY, USA). The Fiji software [26] was used to quantify the fluorescence intensity of each slide. The same fluorescence intensity was used to image all the samples and same threshold was used to quantify the fluorescence intensity using Fiji. A total of 8 mice were used per group.

#### 2.11.3. Therapeutic efficacy of NPs in vivo

The therapeutic efficacy of the NPs was determined by giving the 2 mg/ml NP suspension and free drugs (free INT: 1 μM, free DEX: 2 μM) into mice at predetermined time intervals. Following the 10-day EtOH diet, all the mice were subjected to cheek pouch procedure to collect blood. At day 10, around 100 μl of blood was collected. At day 11, mice were injected with NPs or free drug intraperitoneally. 200 μl of 2 mg/ml NP suspension or free drugs in PBS were given every three days up to 25 days. During the study period, 5 intraperitoneal (IP) injections were given. The study groups consist of eight total groups, including control (control diet-fed), ALD control (5 % EtOH diet-fed), free INT, free DEX, PC-1 NPs, PC-1+INT NPs, PC-1+DEX NPs, PC-1+DEX + INT NPs. The second cheek pouch procedure was carried one week after the first procedure. On day 25, all mice were euthanized, and blood was collected. Harvested organs were processed similar to the biodistribution study. A total of seven mice were used per group.

#### 2.11.4. Liver enzymes and pro inflammatory cytokine production in vivo

The collected plasma from the treatment study was used to detect the AST and ALT liver enzymes and pro-inflammatory cytokines such as IL-1β, TNF-α, and IL-6. The AST ELISA kit (Abcam, ab263882, Boston, MA, USA), and ALT ELISA kit (Abcam, ab282882) to detect the AST and ALT liver enzymes levels in mouse according to the manufacturer’s instructions. The IL-1β ELISA (BioLegend, 432604, San Diego, CA, USA), TNF-α ELISA (BioLegend, 430904), and IL-6 ELISA (BioLegend, 431304) were used to evaluate the therapeutic effects of the NP system on proinflammatory cytokine production as outlined in manufacturer’s protocols.

#### 2.11.5. Histological evaluation and immunohistochemistry (IHC)

Hematoxylin and eosin (H&E) staining was used to evaluate the histological features of liver samples treated with different NP groups and free drug. All the other organs including spleen, kidney, lung, heart, and brain were also stained with H&E to assess if there is any NP-induced inflammation in these organs.

Lipid droplet formation in liver due to EtOH diet was examined using Oil red O (ORO) staining. The frozen unfixed tissues were used for this study. The tissues were first fixed in 10 % formalin solution and followed by the manufacturer’s instruction. After ORO staining, tissues were counterstained with hematoxylin and covered with coverslip using water based mounting media. The tissue sections were observed using EVOS light microscope and Oil droplets further quantified using Fiji software.

The F4/80, a KC-specific marker, was used to evaluate the targeting efficacy of the NPs by immunohistochemistry. An overlap of NP fluorescence with the F4/80 fluorescence was expected to confirm NP specificity. Rabbit anti F4/80 primary antibody (Abcam, ab300421) and goat anti-rabbit Alexa Fluor 568 secondary antibody (ab175471) were used in the standard IHC protocol in frozen biodistribution study liver tissues.

Cytochrome P 450 family 2E1 (CYP2E1) enzyme plays a critical role in alcohol metabolism and is known to be elevated in ALD. Therefore, CYP2E1 expression of the NP treatment groups were detected using rabbit anti CYP2E1 primary antibody (ThermoFisher, PIMA532605, Waltham, MA, USA) and goat anti-rabbit Alexa Fluor 568 secondary antibody (ab175471).

### 2.12. Statistical analysis

All the experiments were conducted independently in triplicates or quadruplicates unless otherwise mentioned and results were expressed as mean ± standard error. Statistical analysis (two-way ANOVA) was performed with the help of GraphPad Prism software. Statistical significance was indicated as *p < 0.05, **p < 0.01, ***p < 0.001, ****p < 0.0001. For *in vivo* studies, the number of mice used per treatment group was determined based on power calculations to detect meaningful differences with a power of 0.80 at α = 0.05 and assuming an effect size (d) of 1.0.

## 3. Results and discussion

Despite the high prevalence of ALD worldwide, there are limited effective treatment options available, especially during its advanced stages. Traditional therapeutic approaches often face challenges such as poor pharmacokinetics and significant off-target effects due to systemic administration [1,27]. Current liver-targeting NP systems primarily focus on hepatocytes by utilizing receptors such as asialoglycoprotein and glycyrrhizin, as well as targeting HSCs and liver sinusoidal endothelial cells [28,29]. In comparison, there have been relatively few studies targeting KCs despite their critical role in liver disease progression. Furthermore, most of the KC-focused research involve targeting the mannose receptor [30], which is expressed by several different types of cells leading to reduced specificity. Furthermore, most studies have emphasized the inhibition of KCs rather than directly targeting them for treatment [31,32]. To our knowledge, this is the first study to target the Gpbar1 receptor expressed only by KCs in the liver, for liver-specific drug delivery to treat alcohol-associated inflammation.

### 3.1. Characterization of NP diameter and surface coating

**Fig. 1.**
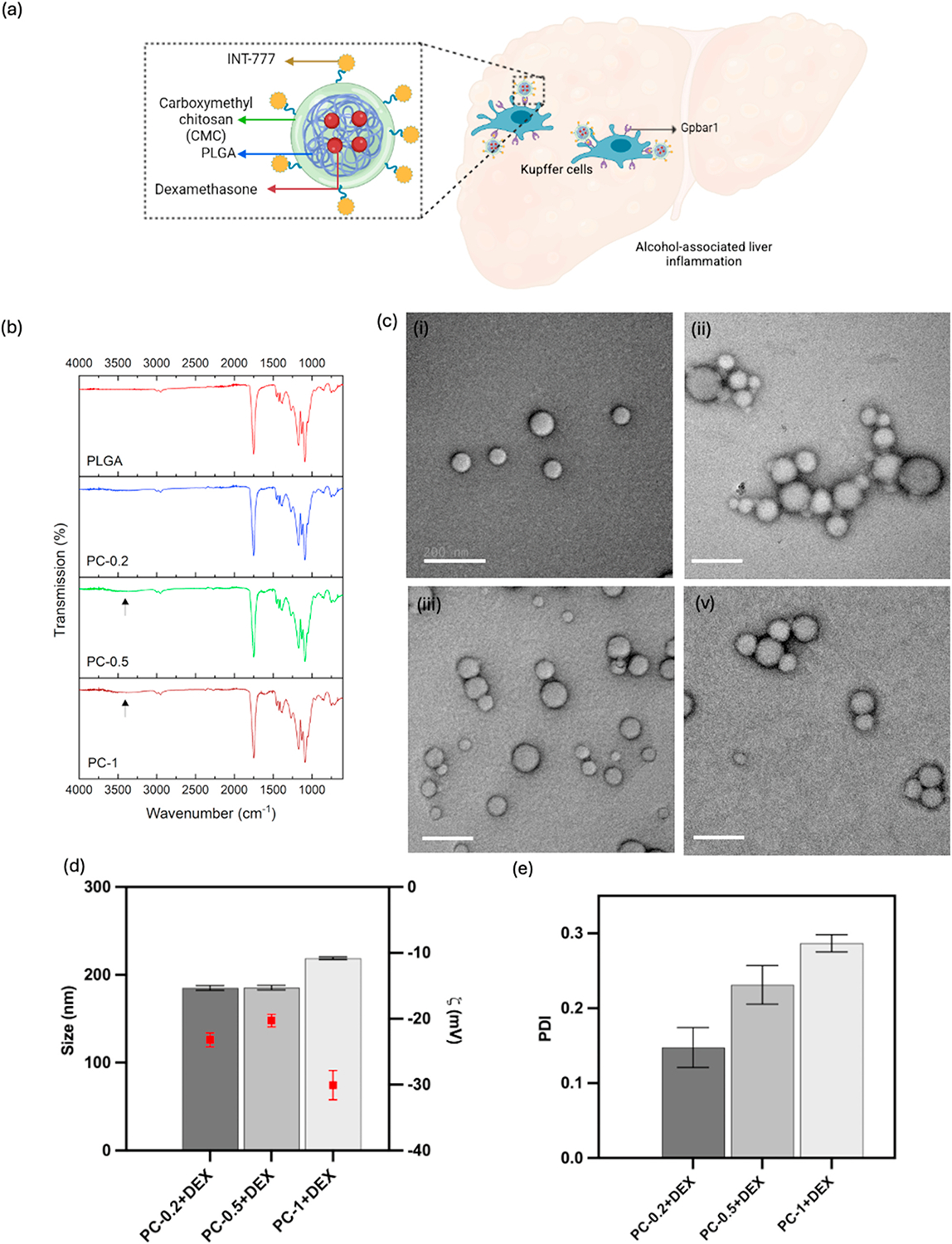
**(a)** Schematic representation of NPs targeting Gpbar1 receptors in KCs using INT-777- conjugated NPs; **(b)** FTIR spectra of NPs with different compositions of CMC, confirming incorporation of both PLGA and CMC in the final formulation; **(c)** TEM images of NPs: (i) PLGA only, (ii) PC-0.2, (iii) PC-0.5, (iv) PC-1 (scale bar = 200 nm) confirming relatively uniform, spherical morphologies; **(d)** hydrodynamic diameter and zeta potential of NPs with DEX and different CMC concentrations **(e)** polydispersity index of the formulated NPs.

The PLGA + CMC (PC) NPs reported in this study consisted of a PLGA core and a CMC shell (Fig. 1a). The modification of PLGA NPs with CMC (core-shell structure) was done to impart stimuli-responsiveness to the system, so that encapsulated therapies can be released in response to the acidic pH environment of the chronically inflamed liver tissue. This pH-responsive property of CMC has been explored for various biomedical applications including targeted drug delivery [33]. The percentage yield of the NPs after CMC coating was in the range of 30–40 % of initial polymer weight. The PLGA NPs with 0.2 %, 0.5 % and 1 % (w/v) CMC, labeled as PC-0.2, PC-0.5, and PC-1 respectively, were successfully synthesized and characterized for their size and morphology. To confirm the CMC coating layer on the NPs, FTIR analysis of PLGA only and the three PC NPs was done. As shown in Fig. 1b, many of the characteristic peaks of CMC overlaps with PLGA, and hence, the hydroxyl group (-OH stretching) peak at around 3450 cm^−1^ was considered as the peak of interest to identify and confirm CMC incorporation. The presence of CMC in the final NP formulation was confirmed by the presence of the OH peak and increasing intensity of the peak with increasing CMC amount, which was not observable in the ‘PLGA NPs only’ group. The characteristic peaks related to PLGA, such as C = O and C–O, were observed at 1743 cm^−1^ and 1072 cm^−1^, respectively. The NP morphology and distribution was visualized using SEM and TEM. As shown in Fig. S1 and 1c, the NPs had spherical shapes with smooth surface. Upon CMC coating, the distinct core-shell structure of the NPs was observed using TEM (Fig. 1c). As indicated in Fig. 1d, the DEX encapsulated NPs showed uniform particles size, where increasing CMC concentrations led to increased diameters; PC-1 NPs had a hydrodynamic diameter of 218.9 ± 0.749 nm compared to 185.1± 1.383 nm and 185.5± 1.309 nm for PC-0.2 and PC-0.5 NPs, respectively. The particles had homogeneous size distribution as confirmed by the narrow PDI of 0.147± 0.013, 0.231± 0.013, and 0.299 ± 0.009 for PC-0.2, PC-0.5, and PC-1 NPs, respectively (Fig. 1e). ZP values of −23.2 ± 0.611 mV, 20.3± 0.555 mV, and −30.1± 1.181 mV, were observed for PC-0.2, PC-0.5, and PC-1 NPs, respectively, indicating excellent stability (Fig. 1d). The higher ZP values may be attributed to the presence of negatively charged deprotonated carboxyl groups of the CMC, as supported by previous research [33].

**Fig. 2.**
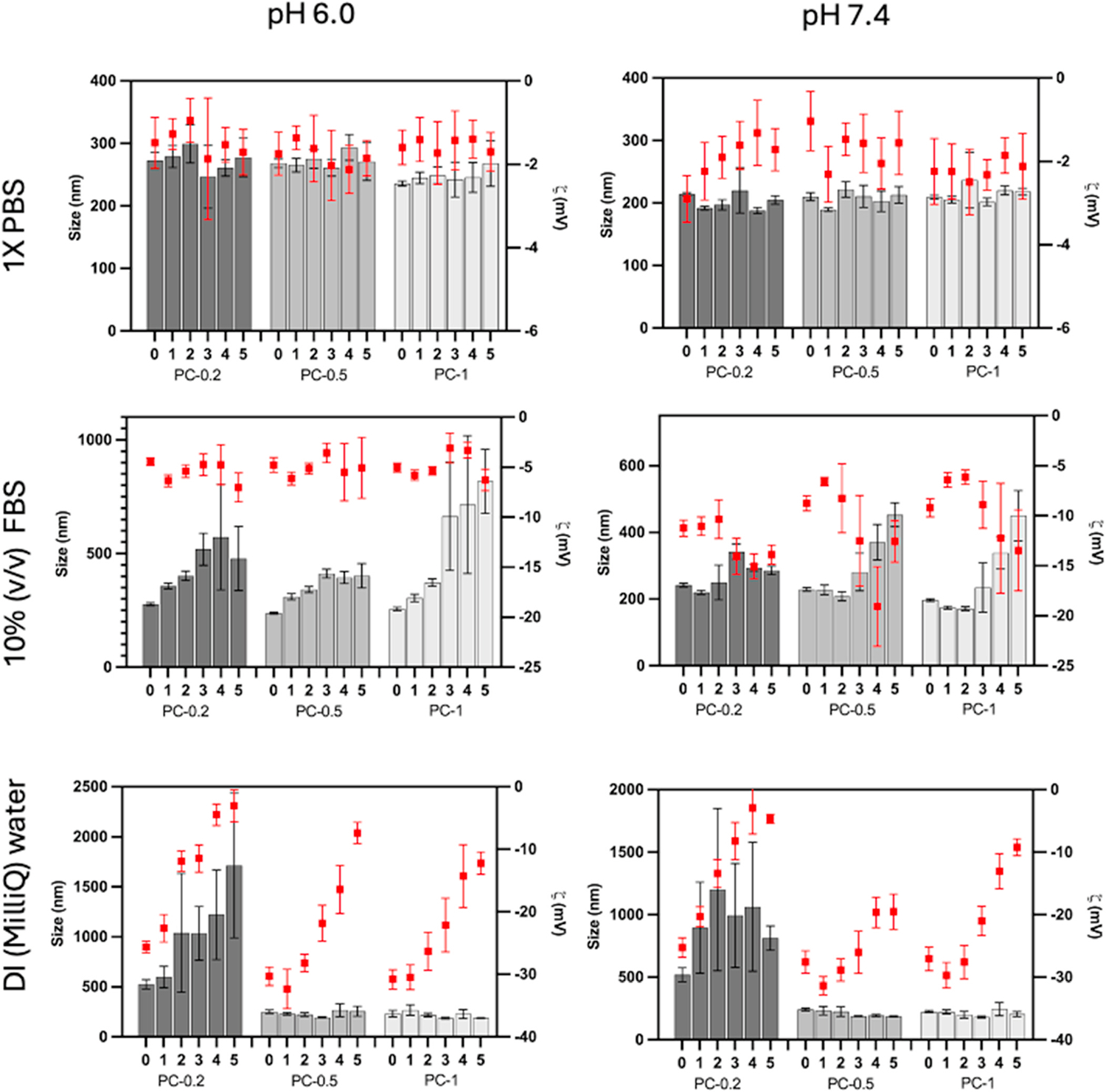
Stability of the NPs at 37°C in 1X PBS, 10 % FBS and DI water at two different pH (6.0 and 7.4), over 5 days; n = 3. The results confirmed that both PC-0.5 and PC-1 NPs are relatively stable in all three solutions, with some size variations in FBS likely due to background interference from proteins in the solution.

We further evaluated the stability of the NPs in three different solutions: 10 % FBS, 1X PBS, and ultrapure deionized (DI) water (see Fig. 2). The NPs remained fairly stable in all tested media for 5 days although PC-0.2 demonstrated increased diameters over time in DI water at pH 6.0 and 7.4. All three NP types showed less than 250 nm size during the five days of the study in pH 7.4 in PBS and also showed stable ZP values. The pH-responsive CMC coating led to an increase in the size of particles at lower pH; this is possibly due to the protonation of the NH_2_ groups, leading to increased repulsive force within the CMC polymer and the resultant swelling. Although the size of NPs increased slightly at pH 6.0 compared to pH 7.4, they remained stable in PBS for 5 days. The NPs in 10 % FBS solution displayed size and zeta potential variation after the first 3 days. This may be mainly attributed to the background reading from albumin and other proteins present in the FBS, and the absorption of these onto the NPs [24]. PC-0.2 NPs, which had the lowest CMC concentration, was unstable in DI water. The high concentration of CMC in the shell of PC-0.5 and PC-1 NPs leads to close packing of the polymer, which prevents rapid swelling. In contrast, the less densely packed shell of PC-0.2 may have absorbed more water, causing it to swell more. From this study, PC-0.5 and PC-1 NPs showed potential for further experiments. The PDI values of the stability study are shown in Fig. S2. Similar to the size variation, pH 6.0 showed slightly increased PDI compared to pH 7.4 (around 0.3) in both PBS and DI solution and as expected 10 % FBS solution showed increased PDI due to the background readings from the proteins. These trends are similar to our previously published PLGA based nanoparticle systems including bare PLGA NPs and modified PLGA NPs [23,24].

### 3.2. PC-1 NPs facilitate pH responsive release of DEX

The encapsulation efficiency of the DEX in PLGA NPs were evaluated using several solvents such as chloroform, acetone, dichloromethane (DCM), before choosing the 1:9 ratio of Methanol to DCM. We analyzed hydrodynamic diameter, PDI, and ZP for each solvent before and after 1 % (w/v) CMC coating as shown in Table S1. Out of 4 different solvent combinations, 1:9 ratio of methanol to DCM showed the highest encapsulation efficiency (6.24 %), which is aligned with other published literature [34]. It was reported that DEX encapsulation in PLGA with DCM as a solvent resulted in 3 % of EE and methanol and acetone are the best solvent for DEX encapsulation [35]. The loading degree of our NP system after CMC (PC-1) coating was 624.92 μg/100 mg of PLGA was much higher than the reported highest loading degree of 226 μg/100 mg of PLGA, showing the high efficiency of our cosolvents in DEX encapsulation.

**Fig. 3.**
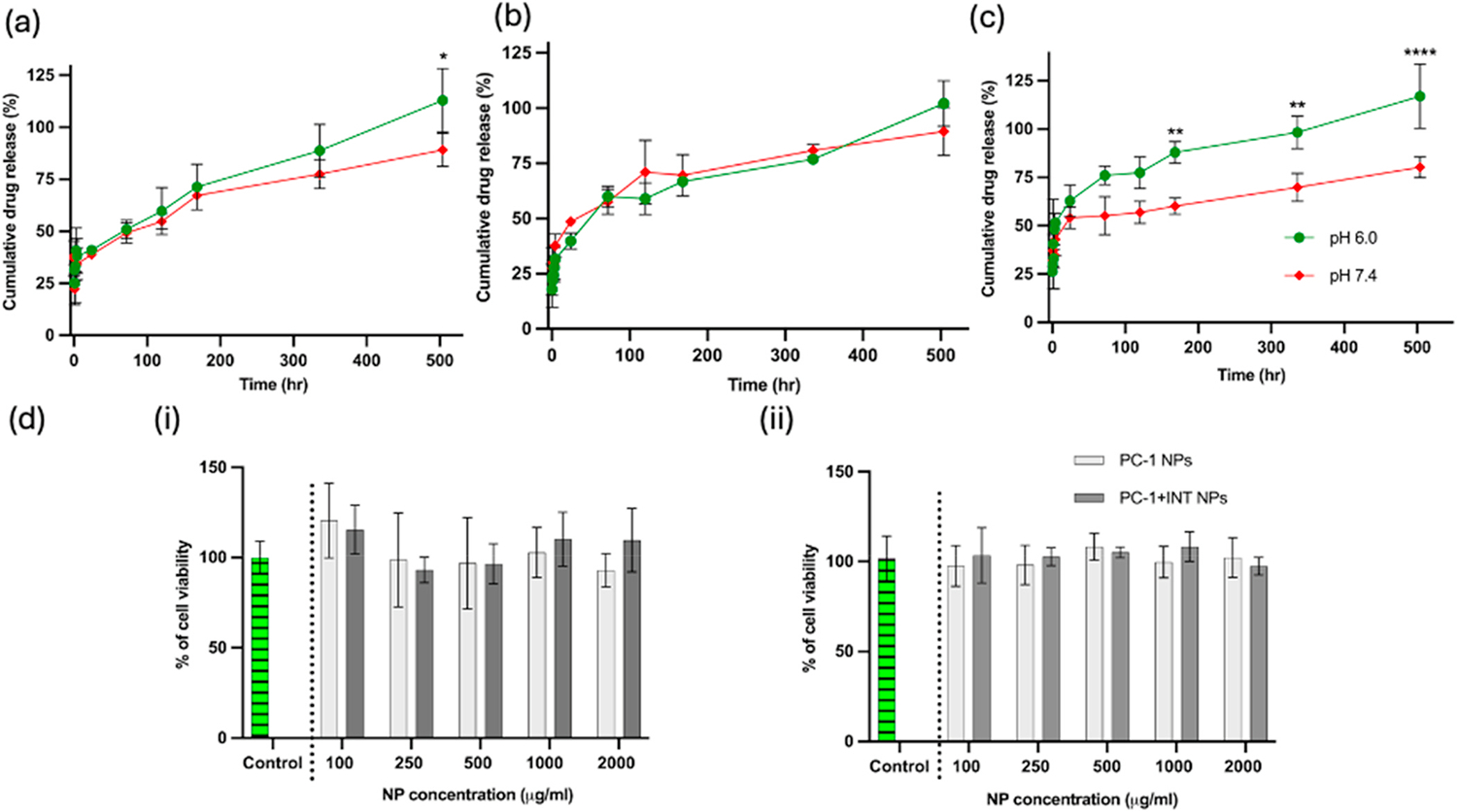
Release of DEX from NPs in 1X PBS for 21 days at 37°C and two different pH: 6.0 and 7.4; **(a)** PC-0.2+DEX; **(b)** PC-0.5+DEX; **(c)** PC-1+DEX: only PC-1+DEX showed significantly high pH-dependent release of DEX; n = 3 **(d)** cytocompatibility studies in (i) HepG2 cells and (ii) DTHP1 (macrophages) confirmed cytocompatibility of PC-1 and PC-1+INT NPs in both cell lines 24 h after treatment; n = 4.

To further evaluate the three NP formulations and choose the most promising formulation for liver-targeted drug delivery, we assessed DEX release in two different pH: 6.0 and 7.4. The DEX released from PLGA was expected to pass through the pH-responsive CMC shell before its release, enabling pH-dependent controlled release [36]. CMC coated PLGA NPs previously developed by us demonstrated greater drug release at pH 6 compared to pH 7.4 [33]. As shown in Fig. 3c, the PC-1 NPs demonstrated distinct differences in the release of the encapsulated DEX at different pH; significantly higher amount of DEX was released at pH 6.0. Both PC-0.2 NPs and PC-0.5 NPs failed to demonstrate similar drastic differences in drug release with changes in pH (Fig. 3a and b). All three CMC concentrations demonstrated almost 100 % drug release during the 21-day study period at pH 6.0. On the third day, 50, 60 and 70 % of the encapsulated DEX was released from PC-0.2, PC-0.5, and PC-1 NPs respectively at pH 6.0, as part of the burst release phase. By day 14, the DEX release percentage was 89, 77 and 99 % for PC-0.2, PC-0.5, and PC-1 NPs, respectively at pH 6.0. In contrast, at pH 7.4, the release on day 14 was 77, 81 and 70 % respectively for the three NP types, highlighting the significant role played by pH in improving drug release from the PC-1 NPs. Our results confirmed that the concentration of CMC in the NPs significantly influenced the drug release based on changes in environmental pH. The pH-responsive CMC shell facilitated a higher release of DEX at a pH of 6.0, which mimics the acidic pH typically found in the ALD microenvironment. Because of the distinct pH-responsive DEX release and excellent stability of PC-1 NPs when compared to the other NPs, it was chosen for further *in vitro* and *in vivo* investigations from this point forward, unless otherwise specified.

### 3.3. In vitro evaluation of the developed NPs

#### 3.3.1. Cytocompatibility and cellular uptake kinetics

HepG2 cells representing hepatocytes and differentiated THP1 monocytes (DTHP1/THP1 macrophages) representing KCs, were used to evaluate the cytocompatibility of NPs at different concentrations ranging from 100 μg/ml to 2000 μg/ml at 24 h post treatment. The cell viability was assessed using metabolic activity-based WST-1 assay. Cells treated with different concentrations of PC-1 and PC-1+INT NPs displayed close to 100 % cell viability, even at the highest concentration of 2000 μg/ml tested, showing the superior biocompatibility of NPs as shown in Fig. 3d. The results from this study are consistent with previous studies conducted by our lab using PLGA and PC NPs [33].

**Fig. 4.**
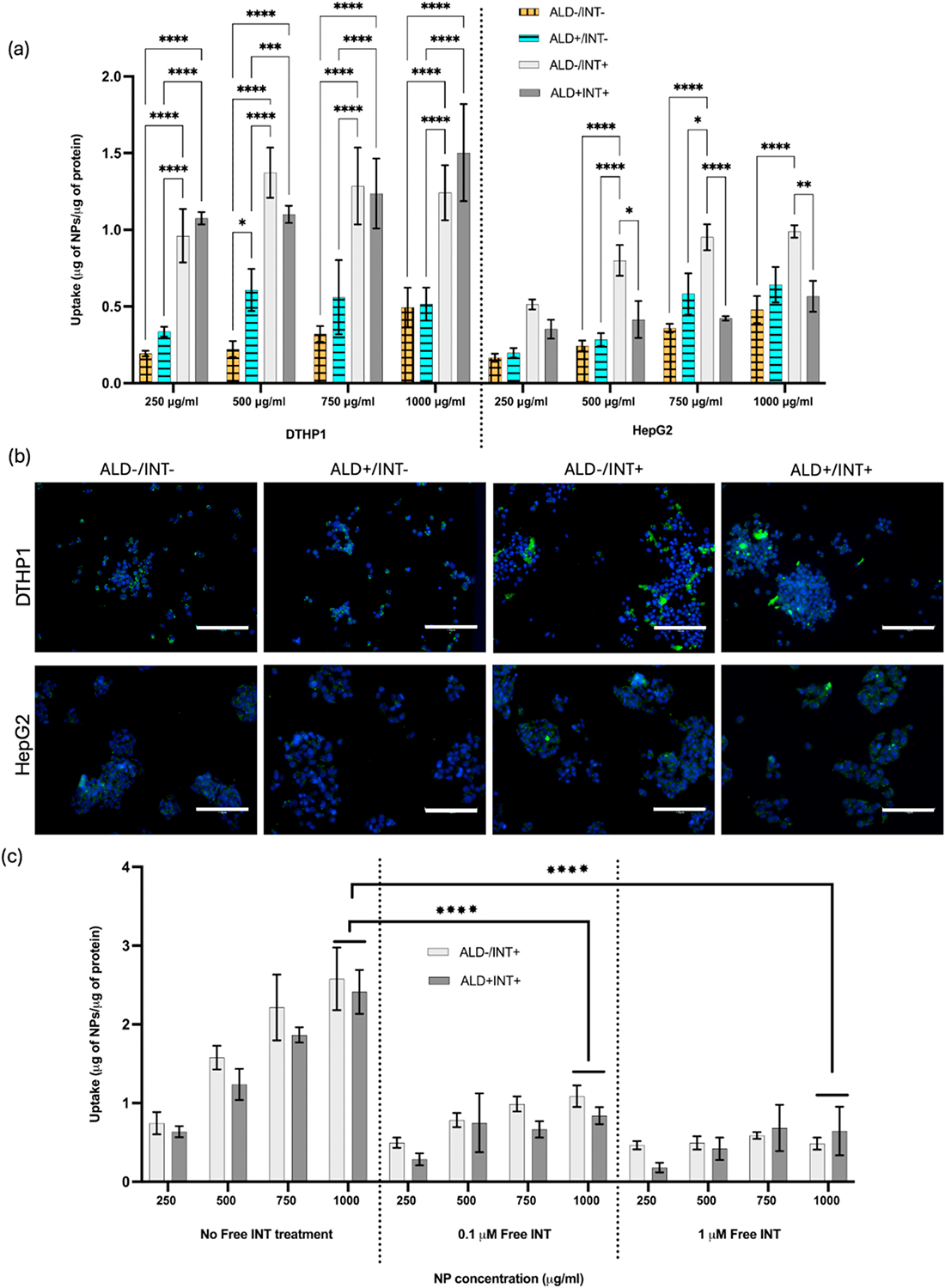
Uptake of NPs by DTHP1 and HepG2 cells treated with 1 μg/ml LPS and 0.16 % (v/v) EtOH; **(a)** quantitative analysis of NP uptake at different concentrations, confirmed higher, dose-dependent uptake by DTHP1 cells.; n = 4 **(b)** fluorescence imaging of NP uptake also confirmed higher uptake in DTHP1 than in HepG2 cells: green-coumarin-6 loaded NPs, blue-nuclei (scale bar = 150 μm) **(c)** quantification of NP uptake after cells were pre-treated with free INT (0.1 μM and 1 μM); uptake of the PC-1+INT NPs reduced due to the free INT pretreatment, confirming Gpbar1 targeting capabilities of the NPs.

The *in vitro* cellular uptake kinetics of the NPs was assessed by using HepG2 and DTHP1 cells. In this study, ALD was replicated *in vit*ro by treating the cells overnight with both LPS and EtOH. Physiologically, drinking alcohol increases the gut permeability, causing endotoxins like LPS to enter the circulation through portal vein [25,37]. Therefore, 1 μg/ml LPS was added to the media containing 0.16 % (v/v) EtOH to replicate ALD. The incubation time of 2h was chosen based on previous studies by our group and others where 2h was found to be optimal for observing dose-dependent uptake kinetics of PLGA-based NPs [38]. Significantly higher uptake of PC-1+INT NPs was observed in the DTHP1 cells regardless of the LPS/EtOH treatment, due to high Gpbar1 expression in these cells. The uptake of NPs by HepG2 cells was significantly lower than that observed the DTHP1 cells. The presence of INT in the NPs had no influence on NP uptake by HepG2 cells due to the absence of Gpbar-1 receptors (Fig. 4a). Fluorescence imaging of uptake of coumarin-6 containing PC-1+INT NPs (250 μg/ml) confirmed higher uptake in the DTHP1 cells than the HepG2 cells (Fig. 4b). The 500 μg/ml NP concentration uptake images are shown in Fig. S3. The similar trend was observed in the fluorescence images as more NPs were uptake by the DTHP1 cells compared to HepG2 cells. The INT conjugated NPs showed more uptake by DTHP1 cells, similar to the quantitative study. The quantification of NPs using Fiji [26] (ImageJ) (Fig. S3b) also provide similar trend as the quantitative analysis. The quantitative data shows that DTHPs pre-treated with 0.1 μM and 1 μM of free INT showed lower uptake of NPs (Fig. 4d). The pre-treatment of free INT for 2 h reduced the PC-1+INT NPs uptake significantly in all concentrations. However, there was no significant difference in NP uptake between groups treated with low and high free INT concentrations. The pre-treated groups showed lower uptake because of the unavailability of Gpbar-1 binding sites as they were already bound to free INT. This confirmed the selective targeting of Gpbar-1 receptor using INT-777.

#### 3.3.2. Effects of PC-1+DEX + INT NPs on pro-inflammatory cytokine release

As a result of chronic alcohol consumption, the liver is exposed to excess gut-derived bacteria and bacterial components, such as LPS. When excess LPS is present in the liver, toll-like receptors (TLRs) in KCs are activated and produce proinflammatory cytokines, including tumor necrosis factor-α (TNF-α) and interleukins such as IL-1β and IL-6 [31, 39]. TNF-α plays a significant role in developing ALD-related inflammatory responses, including the development of acute septic shock and hepatocyte death. To assess the role of the developed NPs in suppressing proinflammatory cytokine release, DTHP1 cells were concurrently treated with 1 μg/ml LPS with 0.16 % (v/v) EtOH, and free drug or the different NP formulations as previously described, and cytokine release was assessed 6h and 24h following treatment. Free INT two doses were selected as 0.1 μM and 1 μM, while free DEX two doses were selected as 0.2 μM and 2 μM according to the release profile of DEX and conjugated concentration of INT corresponding roughly to the amounts on or released from the NPs during the course of the experiment. Similarly, all the NP concentrations were selected as 500 μg/ml and 1000 μg/ml for low and high doses, respectively.

**Fig. 5.**
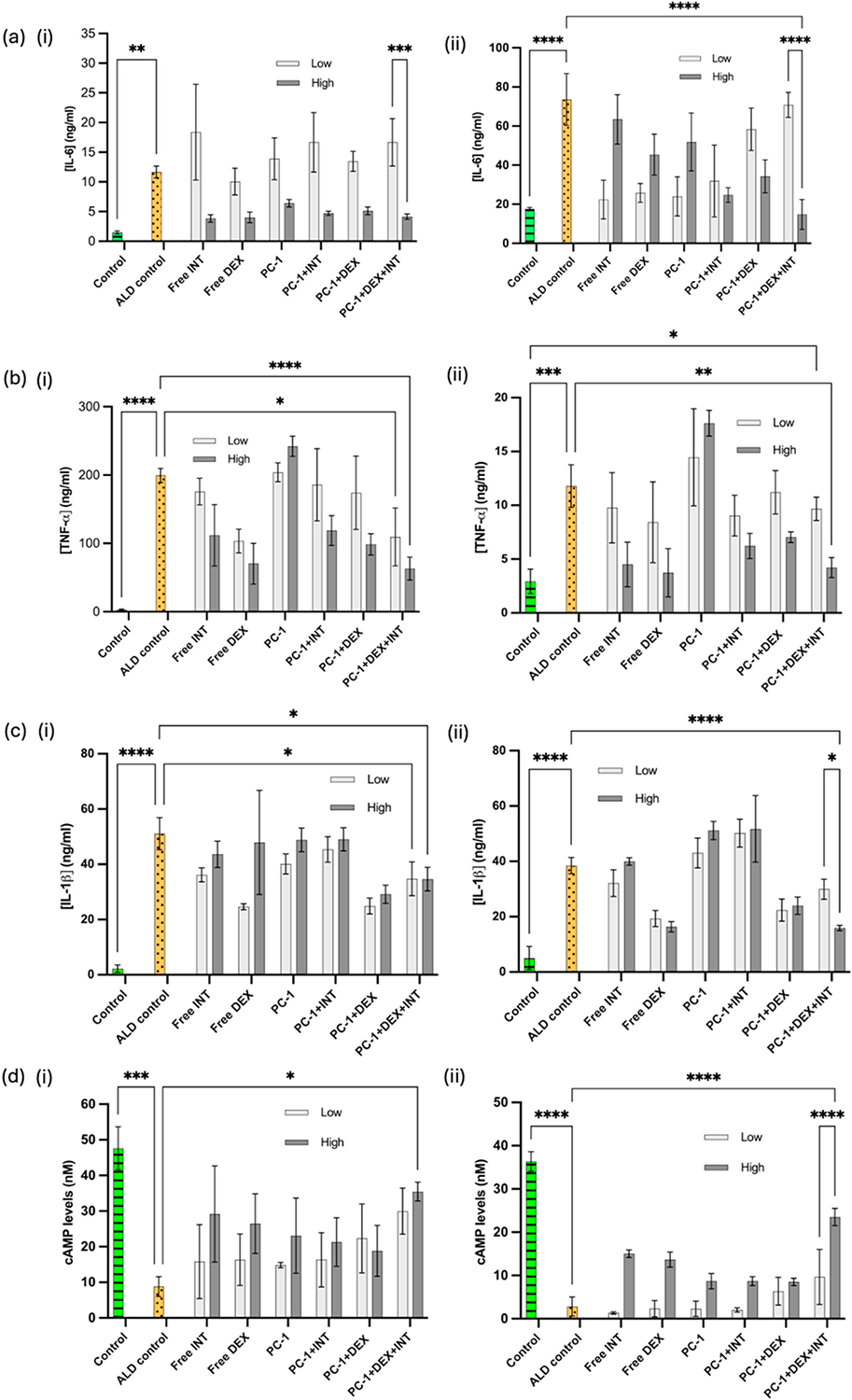
Cytokine and cAMP assays on DTHP1 cells. **(a)** IL-6 activity; **(b)** TNF-α activity; **(c)** IL-1β activity, and **(d)** cAMP activity of cells after treatment with different free drug and NP concentrations at (i) 6 h and (ii) 24 h of treatment: all the groups except control were treated with 1 μg/ml LPS and 0.16 (v/v)% EtOH and same time treated with Free INT (low-0.1 μM, high- 1 μM), Free DEX (low-0.2 μM, high- 2 μM), PC-1, PC-1+INT, PC-1+DEX, and PC-1+DEX + INT (low- 500 μg/ml, high-1000 μg/ml NP concentrations). Decreased cytokine levels and increased cAMP levels were observed for the PC-1+DEX-INT NPs at the higher concentration, particularly at 24 h.

Studies show that IL-1β and TNF-α expressions tend to be maximum at the first 3 h when exposed to inflammatory compounds such as LPS and EtOH, followed by gradual decrease in expressions.

over time, while IL-6 levels increase up to 24 h after treatment [40, 41]. As shown in Fig. S6, overnight exposure of DTHP1 cells to LPS/EtOH followed by treatment with the NP formulation did not provide the expected results possibly due to the inherent challenges associated with maintaining cytokine expression levels *in vitro*. Therefore, co-treatment of cells with NPs and LPS + EtOH and assessment of NP effects at 6h and 24h post-treatment was done. IL-6 shows ambivalent effect on the liver, and may act as both pro- and anti-inflammatory cytokine depending on the stimulus [42–44]. The role of IL-6 in ALD is not entirely clear [45], and some studies suggest that IL-6 acts as a pro-inflammatory cytokine similar to other chronic liver diseases, such as MAFLD and HCC [44]. In our study, the final NP formulation, PC-1+DEX + INT, reduced the IL-6 levels in DTHP1 in a dose-dependent manner in the first 6 h, as shown in Fig. 5bi. At 24 h, IL-6 levels were significantly reduced compared to the ALD control in a dose-dependent manner and almost similar to control levels, as shown in Fig. 5bii. TNF-α plays a key role in ALD progression as several studies confirmed elevated TNF-α levels in patients with ALD and higher cytokine levels represent an advanced disease stage [46]. Similar to the IL-6, TNF-α levels were reduced in a dose-dependent manner as shown in Fig. 5ci, ii. As previously mentioned, IL-1β release is directly correlated with alcohol consumption [45]. It also inhibits the liver regeneration [47] and acts as an early damage mediator [48], where expression diminishes overtime. PC-1+DEX + INT groups showed dose-dependent reduction in the IL-1β activity, solidifying the efficacy of the developed NP system.

#### 3.3.3. Effects of PC-1+DEX + INT NPs on cAMP activity

The cAMP is an essential second messenger that regulates various intracellular biological pathways [49,50]. Studies on rat models of ALD which were fed alcohol through a permanent intra-gastric cannula for 2 months with intraperitoneal administration of cAMP showed that cAMP prevented the increase in liver fat accumulation caused by alcohol consumption [51]. The authors suggested that the protective effect of cAMP on alcohol-induced hepatic steatosis was partially mediated by its influence on the alcohol-metabolizing enzyme, CYP2E1. Therefore, analyzing cAMP levels after NP treatment *in vitro* is an important experiment to evaluate the efficacy of our NP system. Following concurrent treatment of LPS and EtOH with NPs, cAMP activity of the THP macrophages were evaluated after 6 h and 24 h as shown in Fig. 5a. The results showed that the PC-1+DEX + INT NPs successfully increased cAMP activity of LPS + EtOH - treated DTHP1 cells in a dose-dependent manner at both 6h and 24 h treatment period. As expected, ALD control showed the lowest cAMP activity.

### 3.4. In vivo studies

#### 3.4.1. Development of ALD in mouse model

Several mouse models have been used for the study of alcoholic liver injury, including NIAAA model (chronic EtOH feeding plus a single binge [52,53]), chronic EtOH feeding (4–6 weeks) plus multiple binges, chronic EtOH feeding (4 weeks) plus three binges during the last 3 days of feeding [54]), intragastric infusion (Tsukamoto-French model), and *ad libitum* oral alcohol in drinking water [55]. Acute gavage of a single dose or multiple doses of EtOH has also been used. The NIAAA model consists of 10 days of chronic EtOH diet followed by a single binge at the day 10. The 10 day chronic EtOH feeding with a single binge and chronic EtOH feeding for 4 weeks followed by three binges during last three days showed significantly elevated ALT levels, but the latter is associated with high mortality rate [52,54]. Intragastric infusion also showed significant elevation of ALT with high mortality rate. Based on the available literature, we introduced a modified NIAAA model without chronic binges to reduce mortality rate while simultaneously inducing liver inflammation with elevated ALT and steatosis.

The NIAAA mouse model of ALD was successfully developed using Liber-Decarli-5 % (v/v) EtOH diet for 10 days as previously described [52]. Then the AST and ALT levels of the mice were analyzed at day 11 to confirm the ALD. The results showed higher AST and ALT levels in EtOH diet-fed mice group compared to control-diet fed mice (Fig. S8b and c). After confirming ALD development, we kept the 5 % (v/v) EtOH diet for another 15 days (total 25 days) to assess the therapeutic efficacy of our NP system. The change in body weight of the mice in each group were recorded with respect to the first day of EtOH diet (Fig. S8d).

#### 3.4.2. Biodistribution study and KC-targeting of NPs

**Fig. 6.**
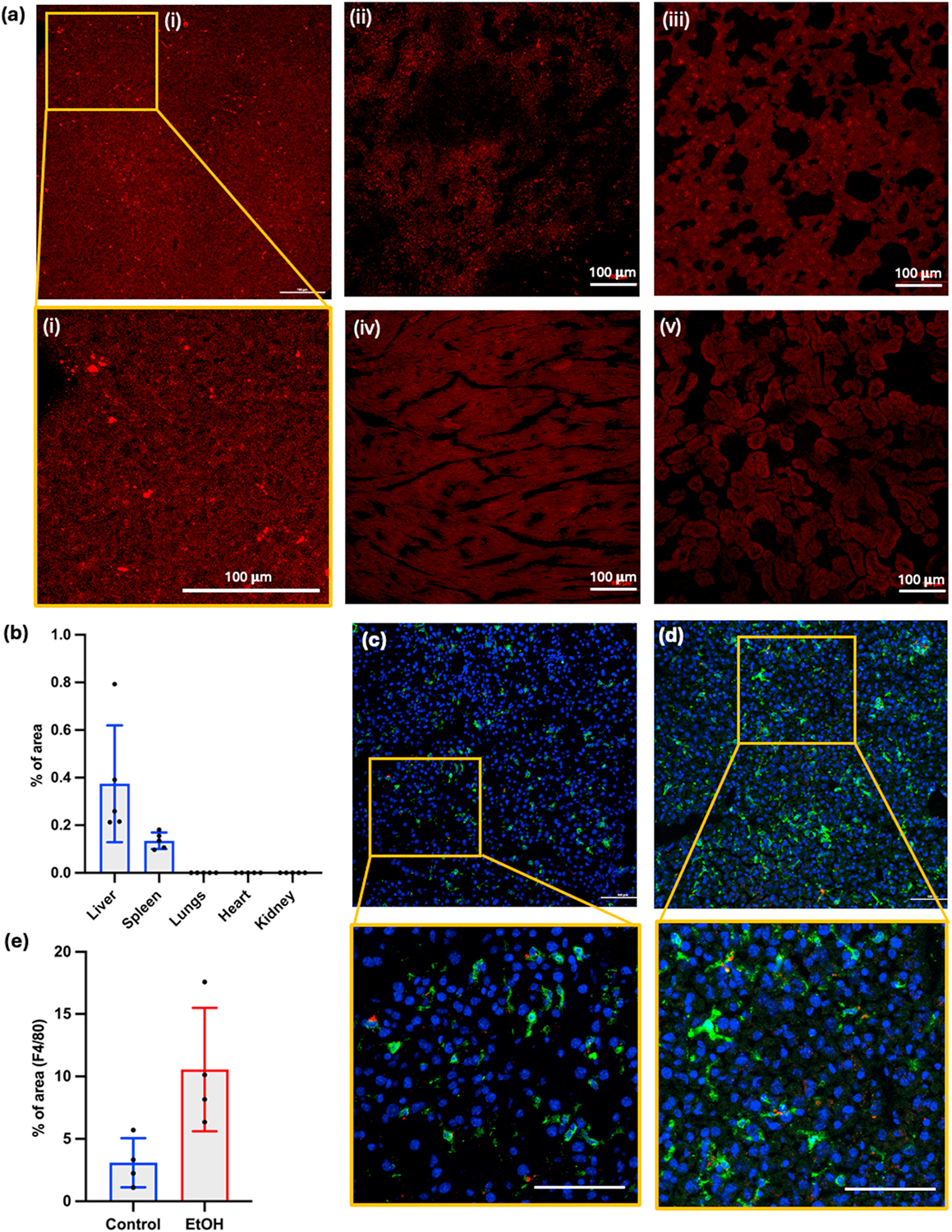
Biodistribution of NPs 24 h post injection: **(a)** NP (red) distribution in (i) Liver; (ii) Spleen; (iii) Lung; (iv) Heart; (v) Kidney; scale bar = 100 **μ**m **(b)** Quantification of NPs using ImageJ in each organ with respect to red fluorescence area (n = 8); **(c)** control diet-fed mice liver image showing F4/80 marker (Kupffer cell specific) around the cells and NPs accumulated around the F4/80 marker suggesting Kupffer cell specific uptake: red- NPs marker, green- F4/80 marker, blue-nuclei; **(d)** EtOH diet fed mice liver showing more F4/80 activity; Scale bar = 100 μm; **(e)** quantification of F4/80 marker/activity in control diet fed and EtOH diet fed mice livers; n = 5; EtOH enhances the F4/80 activity.

After 10 days of EtOH diet, a biodistribution study was carried out to assess the localization of NPs in different organs *in vivo*. As shown in Fig. 6a, most of the NPs were observed in the liver in first 24 h and some NPs can be seen in spleen and lung but none in other organs including, kidney and heart. The quantified fluorescence area as a percentage is shown in Fig. 6b. Also, we quantified the NP accumulation after 72 h, as shown in Fig. S7a and b. Similar to 24 h, there were NPs still present in the liver, but little or no NPs were observed in other organs, including spleen, lung, heart, and kidney. The highest accumulation of NPs was observed at 24 h and it was reduced significantly at 72 h, indicating clearance over time, supported by other literature [56,57]. Since NP fluorescence in the liver had diminished at the 72 h timepoint, it was decided to administer the NPs every three days for the *in vivo* therapeutic efficacy study described below. The PC-1 NPs with and without INT did not show significant difference in accumulation in liver though there was a slight increase in INT NP accumulation as shown in Fig. S7c and 7d. The localization of the NPs around the KCs was further assessed using IHC analysis of the F4/80, a KC-specific marker. As shown in Fig. 6c and d, most of the NPs (red) are concentrated around the KCs. The expression of F4/80 (green) increases with EtOH consumption as suggested.

in other studies [58,59], indicating a significant expansion of macrophage (KCs) population. So, the quantification of green fluorescence in the image showed that elevated F4/80 expression in the EtOH diet-fed mice liver without statistical significance (Fig. 6e).

#### 3.4.3. Effects of PC-1+DEX + INT NPs on inflammatory markers

**Fig. 7.**
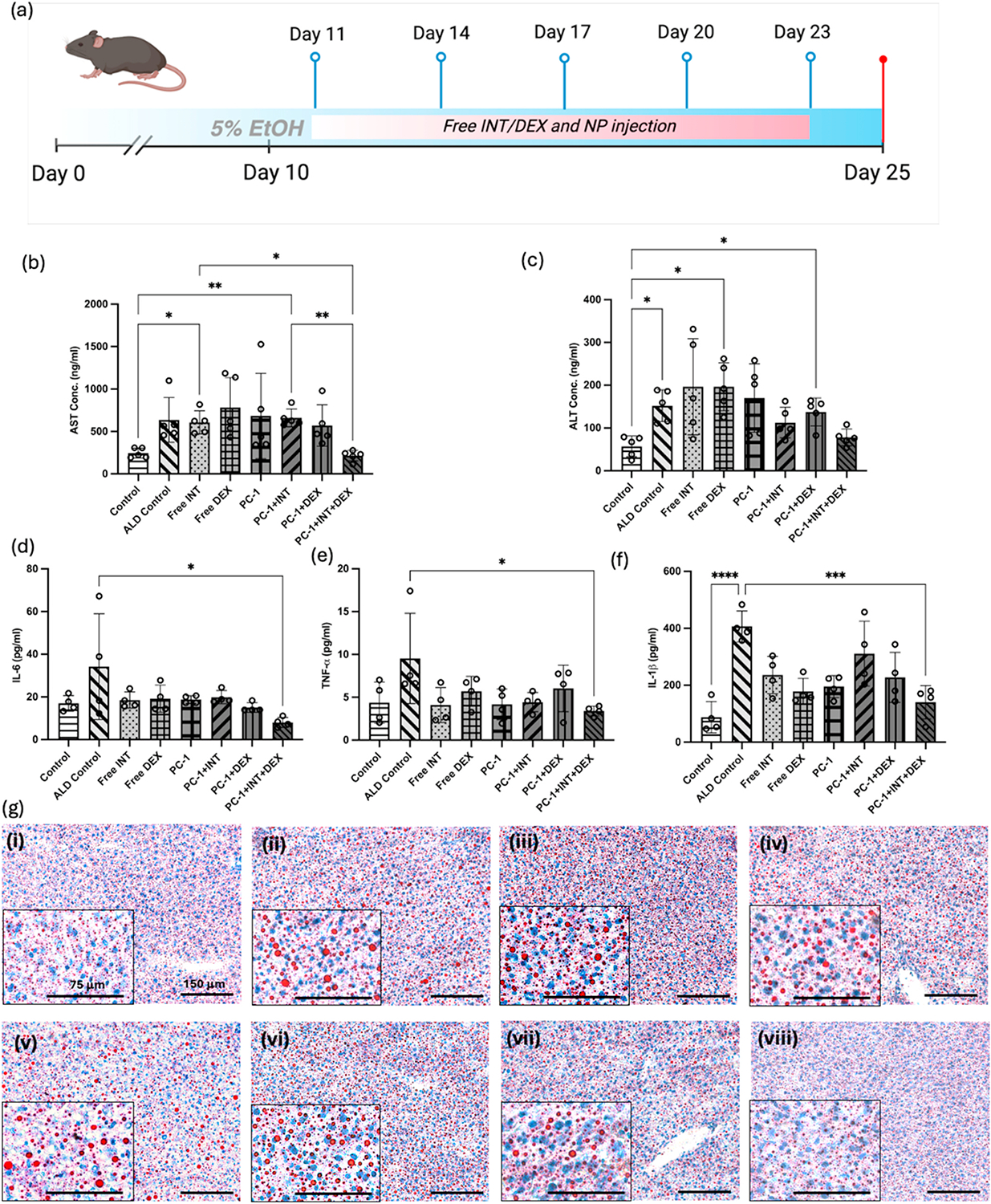
*In vivo* assays **(a)** Schematic representation of therapeutic efficacy study of the NPs using the ALD mouse model; **(b)** Mouse serum AST and **(c)** ALT levels after 15 days of treatment confirmed the significant decrease in both levels following PC-1+INT + DEX NP treatment (Free INT- 1 μM, Free DEX- 2 μM, NP groups- 2000 μg/ml); n = 5; Levels of **(d)** IL-6, **(e)** TNF-**α**, and **(f)** IL-1**β** proinflammatory cytokines after the different treatments also demonstrated decrease after PC-1+INT + DEX NP treatment; n = 4; **(g)** Oil red O images of cryopreserved liver tissues after 15 days of treatments (i) control, (ii) ALD control (5 % EtOH diet), (iii) Free INT, (iv) Free DEX, (v) PC-1, (vi) PC-1+INT, (vii) PC-1+DEX, (viii) PC-1+DEX + INT. Significantly less oil droplets, comparable to the control group, was observed for the PC-1+DEX + INT NP treatment group.

We assessed the therapeutic efficacy of the PC-1+DEX + INT NP system by administering NPs every three days intraperitoneally for a 15-day period. In this study, we tested all the treatment groups that had been tested *in vitro*, including control (liquid control diet fed mice), ALD control, free INT, free DEX, PC-1 NPs, PC-1+INT NPs, PC-1+DEX NPs, and PC-1+DEX + INT NPs. Since 5 % (v/v) EtOH was given to all the seven groups except control, we expected elevated AST and ALT levels in all the groups without DEX. Fig. 7a, b shows the AST and ALT levels of the mice after 15 days of treatment (total 25 days). The group that received the PC-1+DEX + INT NPs exhibited AST and ALT values that were similar to those of the control group. This effect was significant compared to other treatment groups, including the free INT group and the PC-1+INT group. The NPs had a more drastic effect on the AST levels than on the ALT levels. Significant differences in ALT levels were observed between the control group and other ethanol-fed groups, including ALD, free DEX, and PC-1+DEX. On the other hand, the group that received the free drug did not show a reduction in AST and ALT values. This could be due to the rapid clearance of free drugs from the liver within a few hours, which limits their therapeutic ability [60]. Significantly elevated AST and ALT levels indicate the severe status of hepatic injury due to EtOH consumption [61]. This can be achieved in the NIAAA model by giving single dose of 5 % EtOH (the binge consumption) through gavage method and euthanizing after several hours. However, this method could not be implemented in our study because long time survival rate of mice after the gavage was low. We further assessed the pro-inflammatory markers due to the ALD using mouse serum. The IL-1β, TNF-α, IL-6 markers showed elevated activity in the EtOH treated groups compared to control. As shown in Fig. 7c, IL-1β levels significantly reduced in PC-1+DEX + INT group compared to other groups, showing the higher efficacy of the NP system. As shown in Fig. 7d and e, treatment with PC-1+DEX + INT group also led to significant reduction of IL-6 and TNF-α levels compared to the ALD control. To supplement the data obtained thus far, the liver tissue was next assessed for inflammation and lipid droplets using H&E staining and ORO staining, respectively.

**Fig. 8.**
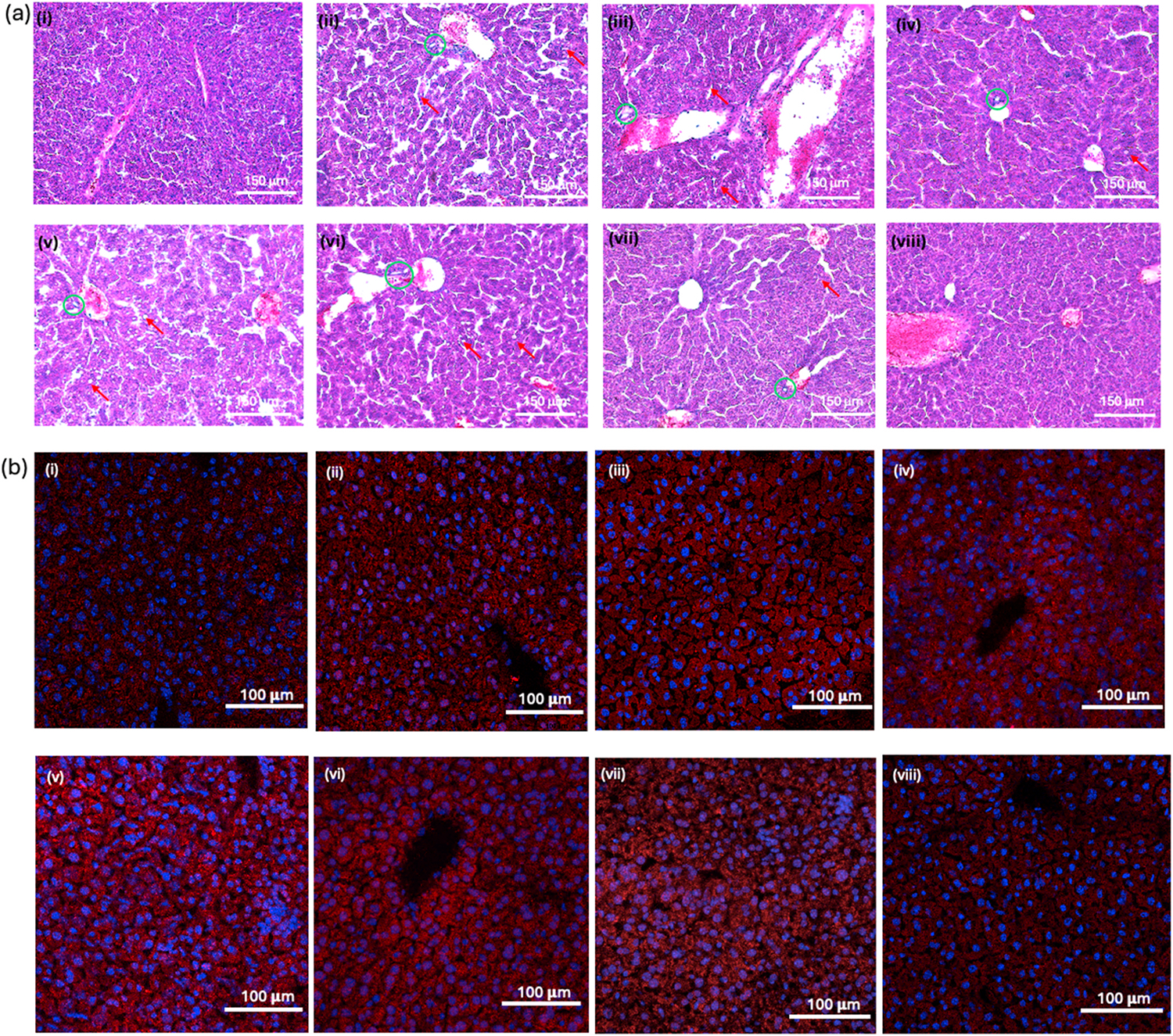
Histology and IHC images. **(a)** H & E images of liver tissues after different treatments (i) control, (ii) ALD control (5 % EtOH diet), (iii) Free INT, (iv) Free DEX, (v) PC-1, (vi) PC-1+INT, (vii) PC-1+DEX, (viii) PC-1+DEX + INT: green circles-macrovesicular steatosis, red arrows-periportal inflammation; **(b)** CYP2E1 activity in cryopreserved liver tissues after 15 days of treatments; (i) control, (ii) ALD control (5 % EtOH diet), (iii) Free INT, (iv) Free DEX, (v) PC-1, (vi) PC-1+INT, (vii) PC-1+DEX, (viii) PC-1+DEX + INT. The PC-1+DEX + INT group had relatively less CYP2E1 activity, which was comparable to the control group.

Fig. 7f shows ORO staining images of liver sections. ORO stains lipid droplets in the liver tissues due to the ethanol induced inflammation. The ALD control and other EtOH diet fed groups showed elevated lipid droplet formation. The control and the PC-1+DEX + INT groups showed the minimal lipid droplet formation, indicating the efficacy of the NP system. Moreover, H&E-stained sections were evaluated to observe the typical inflammation and steatosis morphologies. The ALD control and Lieber-DeCali EtOH diet-fed other groups showed hepatic inflammation such as macrovesicular steatosis and periportal inflammation (Fig. 8a). Macrovesicular steatosis is the most common type of liver disease caused by alcohol. H&E images showed a few well-demarcated fat vacuoles in each hepatocyte, and the nucleus was displaced to the edge of the cell [62]. Periportal inflammation (marked in red circle) occurs around the portal vein of the liver due to the inflammation caused by EtOH and this area surrounded by hepatocytes and KCs. The ALD control, Free INT, PC-1 NPs, PC-1+INT NPs groups showed higher number of macrovesicular steatosis and periportal inflammation. However, the free DEX and PC-1+DEX NPs group showed relatively low inflammation compared to the ALD control and PLGA NP group, showing some improvement because of the anti-inflammatory effects of DEX. As expected, PC-1+DEX + INT NP group significantly reduced inflammation among all groups comparable to control. CYP2E1 is an enzyme that metabolize EtOH/alcohol in the liver [63,64]. Therefore, we assessed the CYP2E1 activity in the liver tissue using IHC method. Fig. 8b shows the fluorescence images of the liver tissues treated with CYP2E1 antibody and Alexa Flora 568 secondary antibody (in red) and nucleus in blue. The control and PLGA + DEX + INT NP group showed the lowest CYP2E1 activity confirmed by the less red fluorescence in these groups when compared to the ALD control and other treatment groups. CYP2E1 is mainly expressed in liver, with hepatocytes showing the highest expression [63]. Some substrates of CYP2E1, such as ethanol, can induce their own metabolism, which is particularly significant for ALD development. Upon activation, CYP2E1 causes oxidative stress by generating reactive oxygen species. Therefore, CYP2E1 inhibition is of interest to prevent ALD progression and liver damage. The significant reduction of CYP2E1 expression in the ALD mouse livers via PC-1+DEX + INT treatment therefore highlights the therapeutic potential of these NPs in the treatment of ALD.

The systemic toxicity from DEX is minimal since the PC-INT NPs can specifically target and accumulate in the liver, as shown in Figs. S9 and 6A at 8h and 24 h post injection respectively. At both timepoints, the NPs predominantly accumulated in the liver, with minimal/negligible accumulation in the spleen and other organs. The DEX dose used in this study was significantly lower than the LD50 (Lethal Dose 50 %) of DEX for a female mouse, which is 794 mg/kg [65]. The DEX released from the injected NPs was less than 40 μg/kg (0.04 mg/kg) in 24 h, which is less than the toxic dose, but the dose was still sufficient to have a combinatorial therapeutic effect with INT-777. The H&E images of other organs also confirmed that there is no toxicity due to nonspecific release of DEX and NP accumulation, as shown in Fig. S10.

## 4. Conclusions

In this work, we successfully developed a novel INT-777 conjugated pH-responsive NP formulation encapsulating the anti-inflammatory drug DEX, for the treatment of inflammation in ALD. The PC-1 NPs demonstrated a significantly higher release of encapsulated DEX at acidic pH, confirming their pH-responsive behavior. The PC-1 NPS and PC-1+INT NPs were cytocompatible with HepG2 and DTHP1 cells. Significantly higher cellular uptake of PC-1+INT NPs by DTHP1 than HepG2 cells was observed regardless of LPS + EtOH treatment, confirming the specificity and targeting capabilities of the NPs. *In vitro* studies confirmed that the PC-1+DEX + INT NPs reduced the production of pro-inflammatory cytokines such as IL-6, TNF-α, and IL-1β by DTHP1 cells. PC-1+DEX + INT NPs significantly increased the cAMP activity in DTHP1 cells when compared to the ALD control. We successfully developed an ALD mouse model using an EtOH ad libitium diet for 10 days, as confirmed by elevated AST and ALT serum levels. The NPs predominantly accumulated in the liver, and were observable up to 72 h post-injection. The IHC study of frozen liver tissues confirmed that NPs were accumulated around the F4/80 marker expressed by KCs in the liver. Serum AST and ALT levels were found to be significantly reduced upon treatment with PC-1+DEX + INT NPs compared to ALD control. The pro-inflammatory cytokines such as IL-6, TNF-α, and IL-1β showed reduced activity compared to ALD control after 15 days of NP treatment. The H&E images confirmed that PC-1+DEX + INT NPs reduced liver inflammation when comparable to the control group, as shown by the reduced macrovesicular steatosis and periportal inflammation. The ORO staining further cemented these findings with a drastic reduction of oil droplets in the PC-1+DEX + INT NP-treated mice comparable to the control. The reduction in CYP2E1 activity also confirmed the efficacy of the NP system in treating ALD. Together, these findings demonstrate the excellent KC targeting and therapeutic capabilities of PC-1+DEX + INT NPs, highlighting their potential for ALD treatment.

## Supplementary Material

1

## Data Availability

Data will be made available on request.

## Appendix A. Supplementary data

Supplementary data to this article can be found online at https://doi.org/10.1016/j.biomaterials.2025.123623.
