# Supplementary material for: A novel Kupffer cell-targeting nanoparticle system to Mitigate alcohol-associated liver disease
Source: Biomaterials. Author manuscript; Available in PMC 2025 Dec 17. (PMC12709622; doi:10.1016/j.biomaterials.2025.123623)
Supplement: 1 [file NIHMS2125489-supplement-1.docx]

**A Novel Kupffer cell-targeting Nanoparticle System to Mitigate Alcohol-associated Liver Disease**

Janitha M Unagolla^1^, Riley Flanagan^2^, Kalindu Perera^1^, Youbin Kim^1^, Curtis Soloff^3^, Emily Kaye^1^, Angela Slitt^1^, Jyothi U Menon^1,2,4,5*^

^1^Department of Biomedical and Pharmaceutical Sciences, College of Pharmacy, University of Rhode Island, Kingston, RI 02881, USA

^2^Department of Chemical Engineering, College of Engineering, University of Rhode Island, Kingston, RI02881, USA

^3^Department of Chemical Engineering, School of Engineering, Worcester Polytechnic Institute, Worcester, MA01609, USA

^4^Legorreta Cancer Center at Brown University, The Warren Alpert Medical School, Brown University, Providence, RI 02912, USA

^5^Department of Biomedical Engineering, College of Engineering, Texas A&M University, College Station, TX, 77843 USA

Supplementary Information


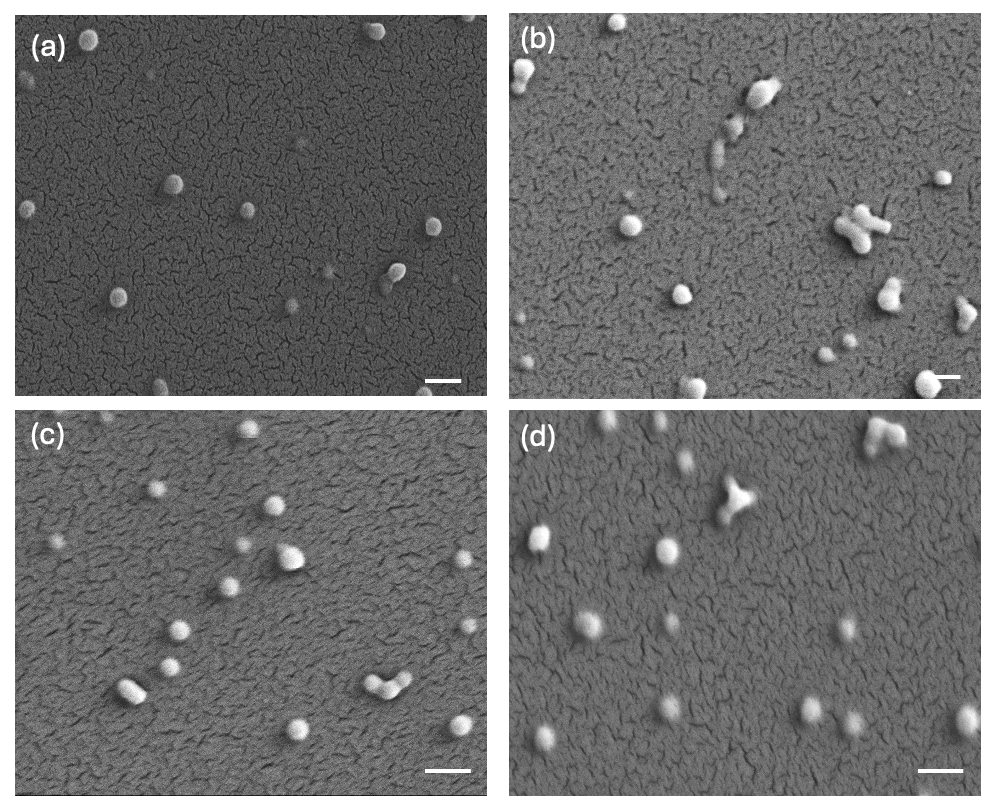


**Fig. S1**: SEM images of NPs: (a) PLGA only; (b) PC-0.2; (c) PC-0.2; PC-1


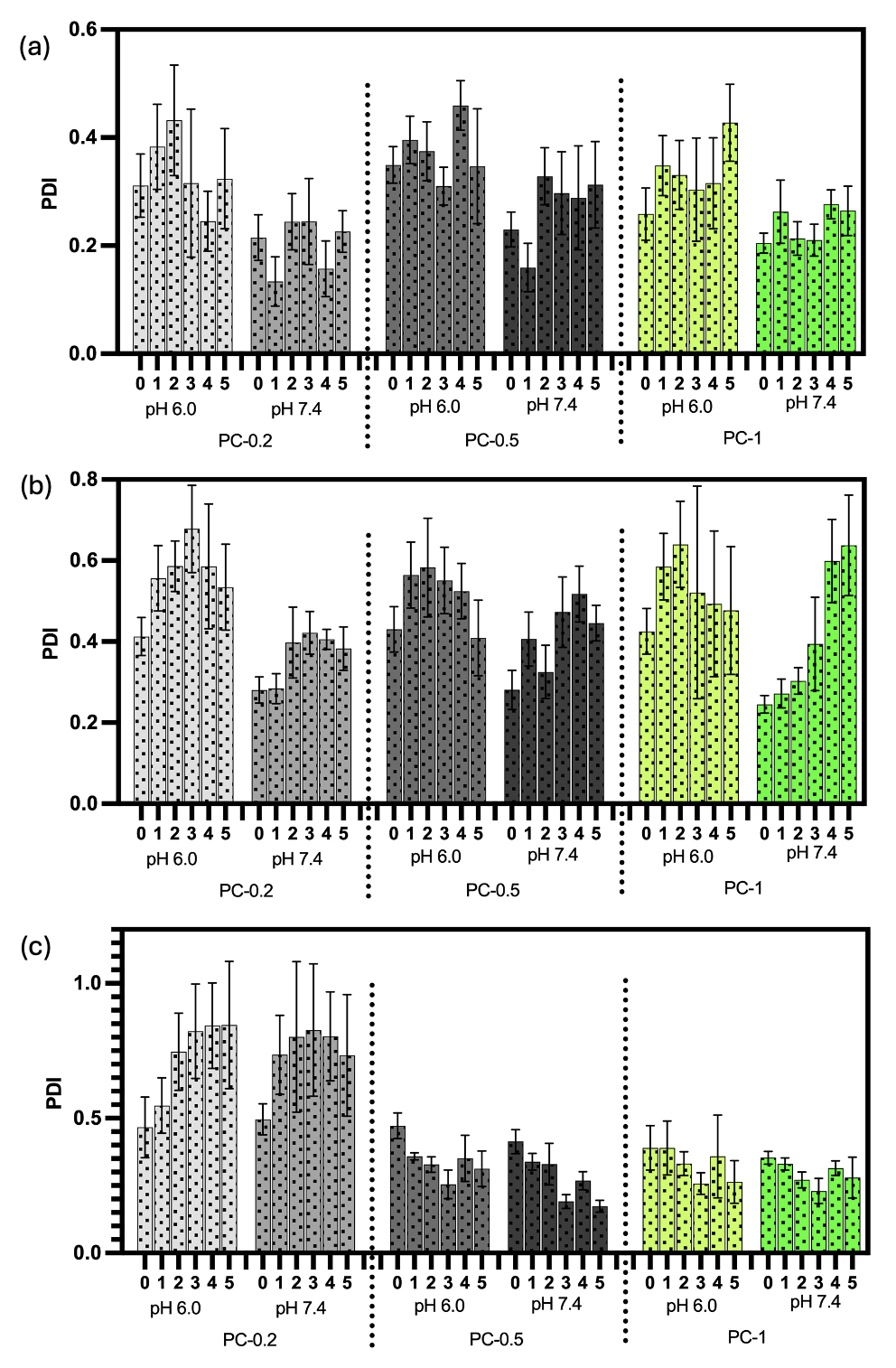


**Fig. S2**. PDI variation of the NPs during stability study from day 0 to day 5: (a) in 1X PBS; (b) in 10% (v/v) FBS; (c) in DI (MilliQ) water


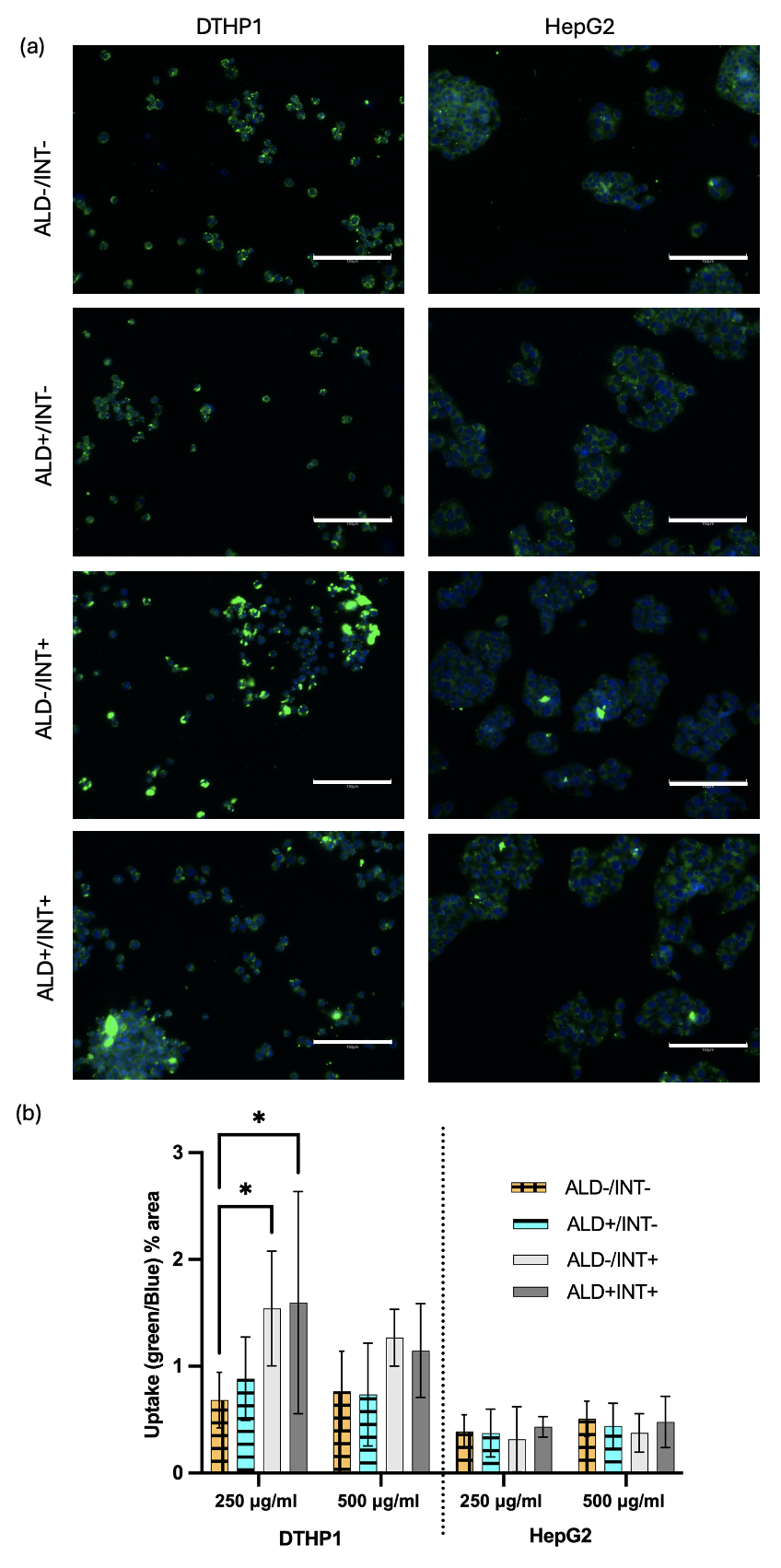


**Fig. S3**. Uptake of NPs part of Figure 4 (b); NP concentration: 500 𝛍g/ml; cells were treated with LPS and 0.16% (v/v) EtOH (ALD+); DTHP1/macrophages shows higher uptake of PC-1+INT NPs compared to HepG2; scale bar: 150 𝛍m


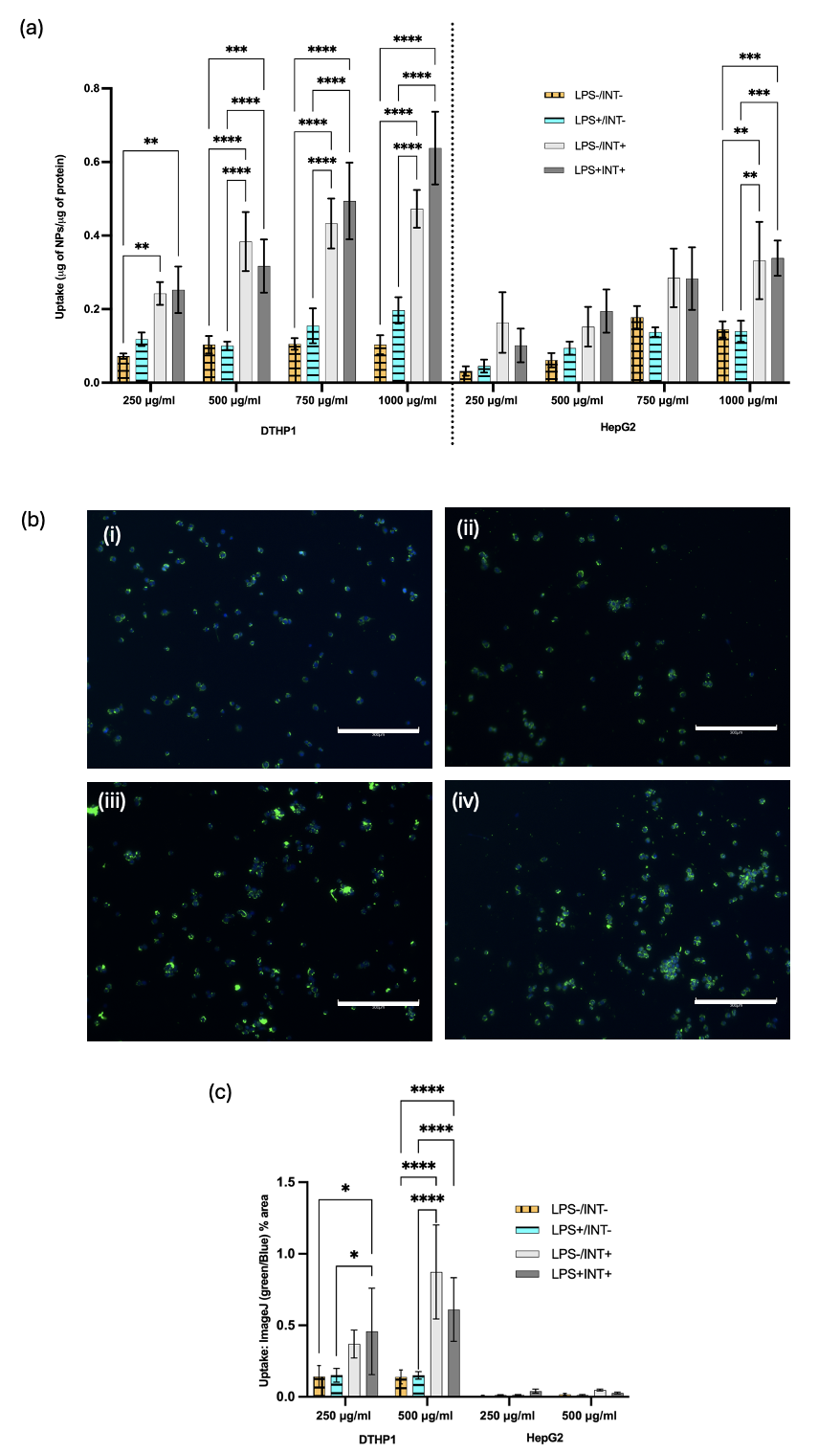


**Fig. S4**. Uptake of NPs after treated with 1 𝛍g/ml LPS only; (a) Quantitative analysis of NP uptake with different concentrations; (b) NPs uptake by DTHP1 in fluorescence images: (i) LPS-/INT-; (ii) LPS+/INT-; (iii) LPS-/INT+; (iv) LPS+/INT-; scale bar: 300 𝛍m (c) Quantification of fluorescence images using ImageJ for two different NP concentrations: 250 𝛍g/ml and 500 𝛍g/ml.


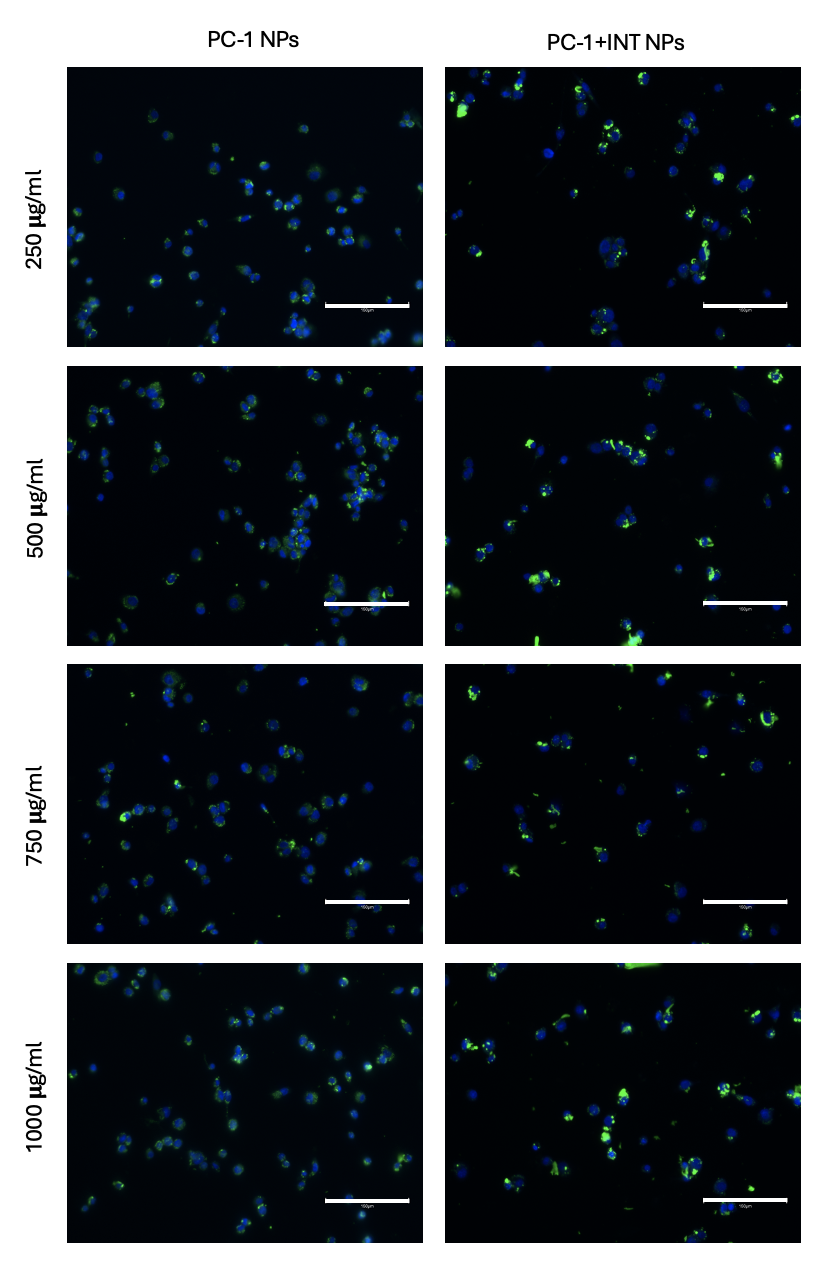


**Fig. S5**. Fluorescence images of NP uptake after free INT treatment (part of Figure 4 (d)); images show higher uptake of PC-1+INT NPs compared to PC-1 NPs; scale bar: 150 𝛍m


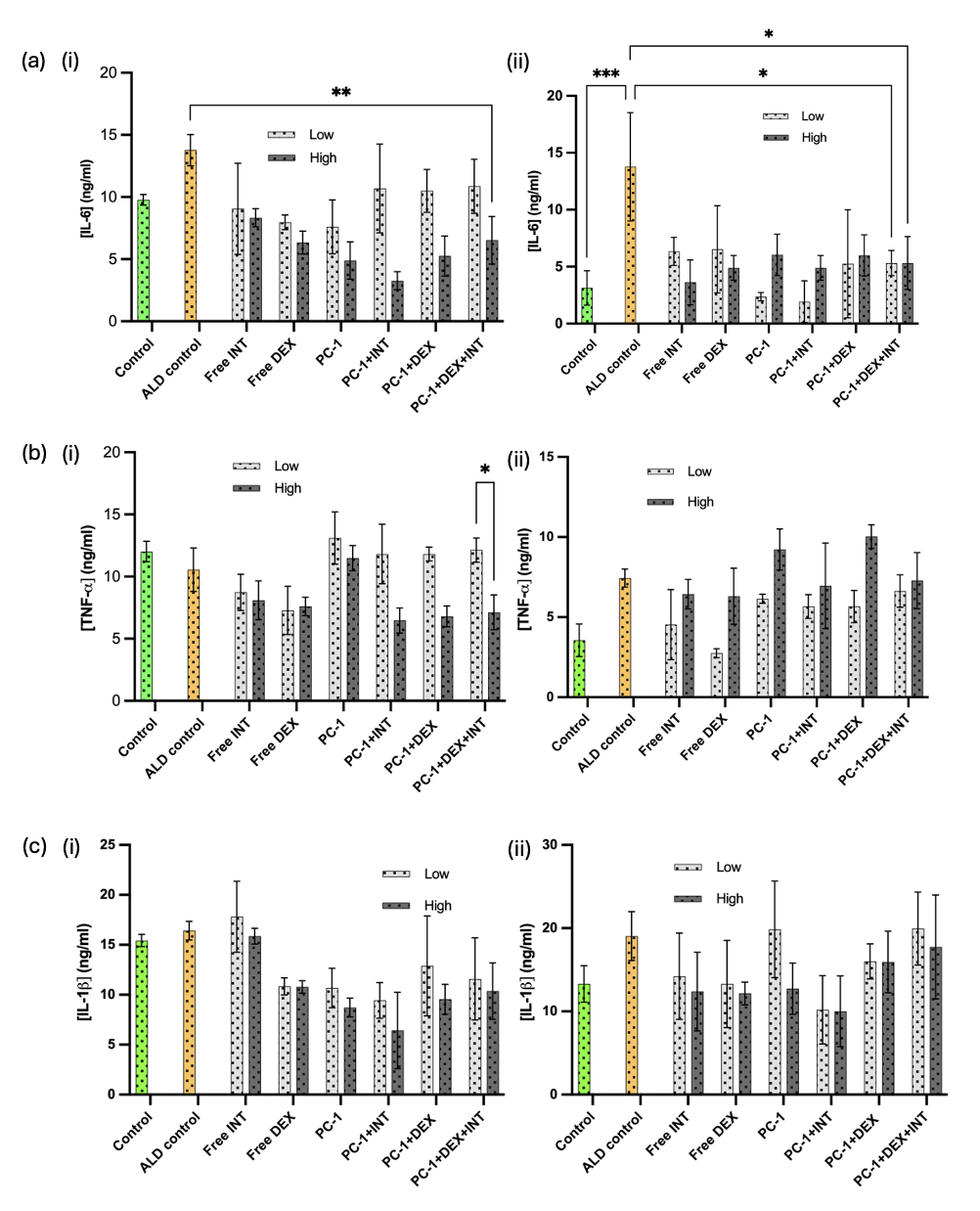


**Fig. S6**.Cytokines activity of the DTHP1 cells where cells were subjected to overnight 1𝛍g/ml LPS and 0.16% (v/v) EtOH treatment followed by NP and free drug treatments (a) IL-6 activity of DTHP1 cells: (i) 2 hr and (ii) 24 hr: treatments are similar to cAMP assay; (b) TNF-𝛂 activity of DTHP1 cells: (i) 2 hr and (ii) 24 hr; (c) IL-1𝛃 activity of DTHP1 cells: (i) 6 hr and (ii) 24 hr; cells treated with Free INT (low-0.1 𝛍M, high- 1 𝛍M), Free DEX (low-0.2 𝛍M, high- 2 𝛍M), PC-1, PC-1+INT, PC-1+DEX, and PC-1+DEX+INT (low- 500𝛍g/ml, high- 1000𝛍g/ml NP concentrations)


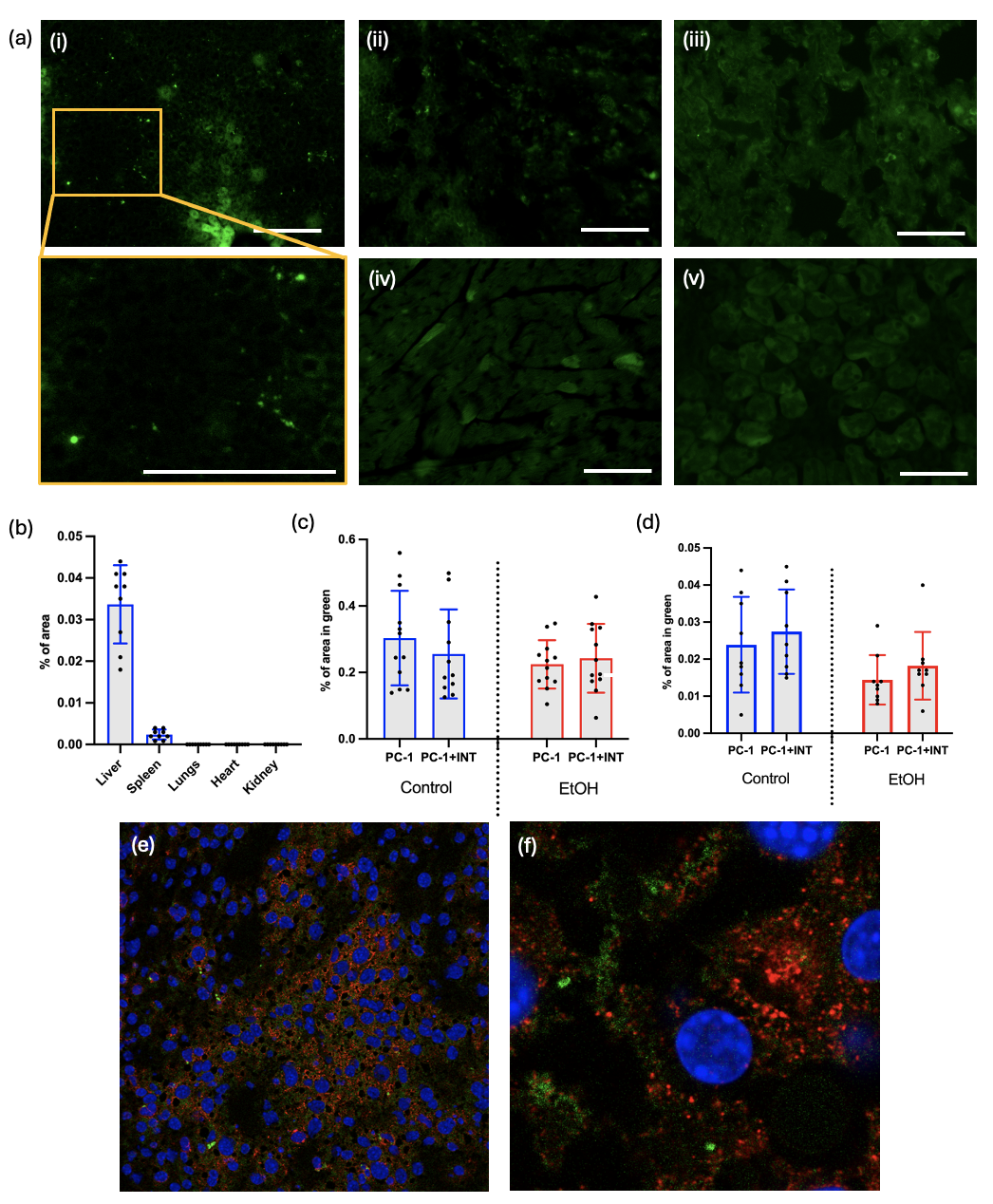


**Fig. S7.** Biodistribution of NPs after 72 hr/ 3 days of post injection: (a) NP distribution in different organs (i) Liver; (ii) Spleen; (iii) Lung; (iv) Heart; (v) Kidney; scale bar- 100 𝛍m (b) Quantification of NPs using ImageJ in each organ with respect to green fluorescence area; (c) Quantification of NPs in liver with respect to different conditions- mice in control diet vs mice in EtOH diet and NPs with INT vs no INT after 24 hrs of post injection (d) Quantification of NPs in liver after 72 hrs of post injection (e) F4/80 marker (Kupffer cell specific) presents around the cells and NPs accumulated around the F4/80 marker suggesting Kupffer cell specific uptake: red- F4/80 marker, green- NPs, blue- nuclei (f) higher magnification of Fig (e).


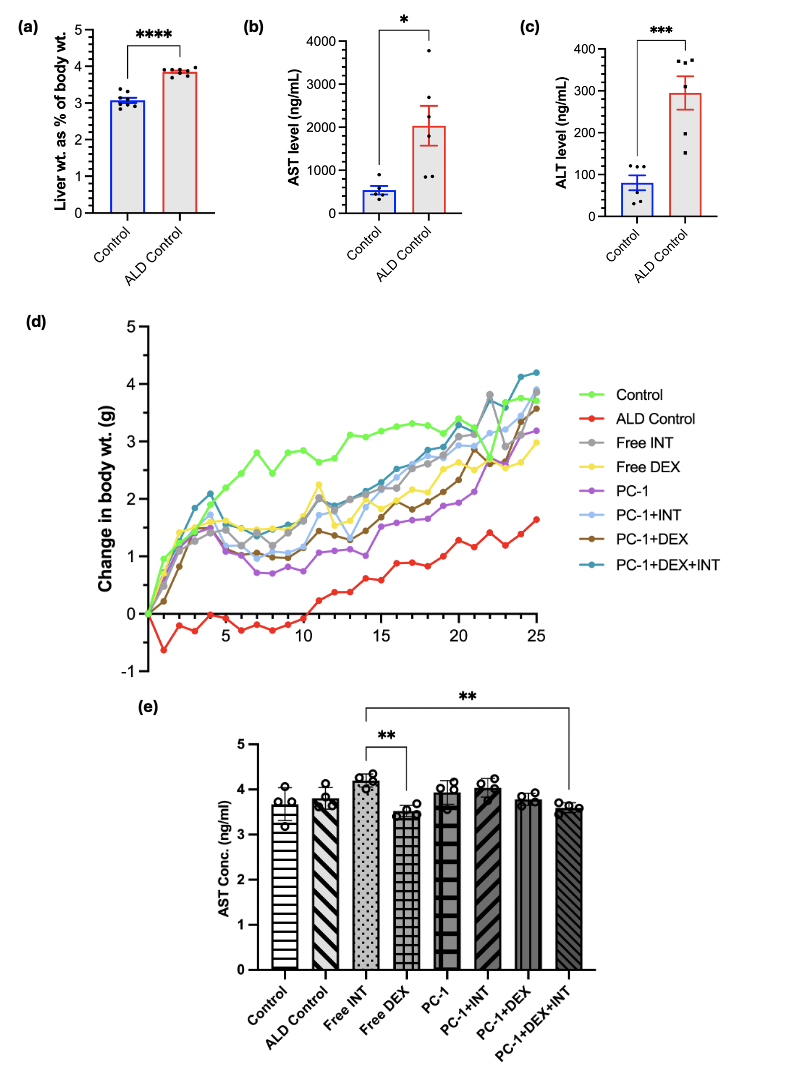


**Fig. S8**: Confirmation of ALD after 10 days of EtOH diet (a) liver weight of mice, control vs EtOH; (b) AST levels; (c) ALT levels; (d) Change in body weight of mice with respect to different treatments; (e) Liver weight of mice as a percentage of body weight with respect to different treatments


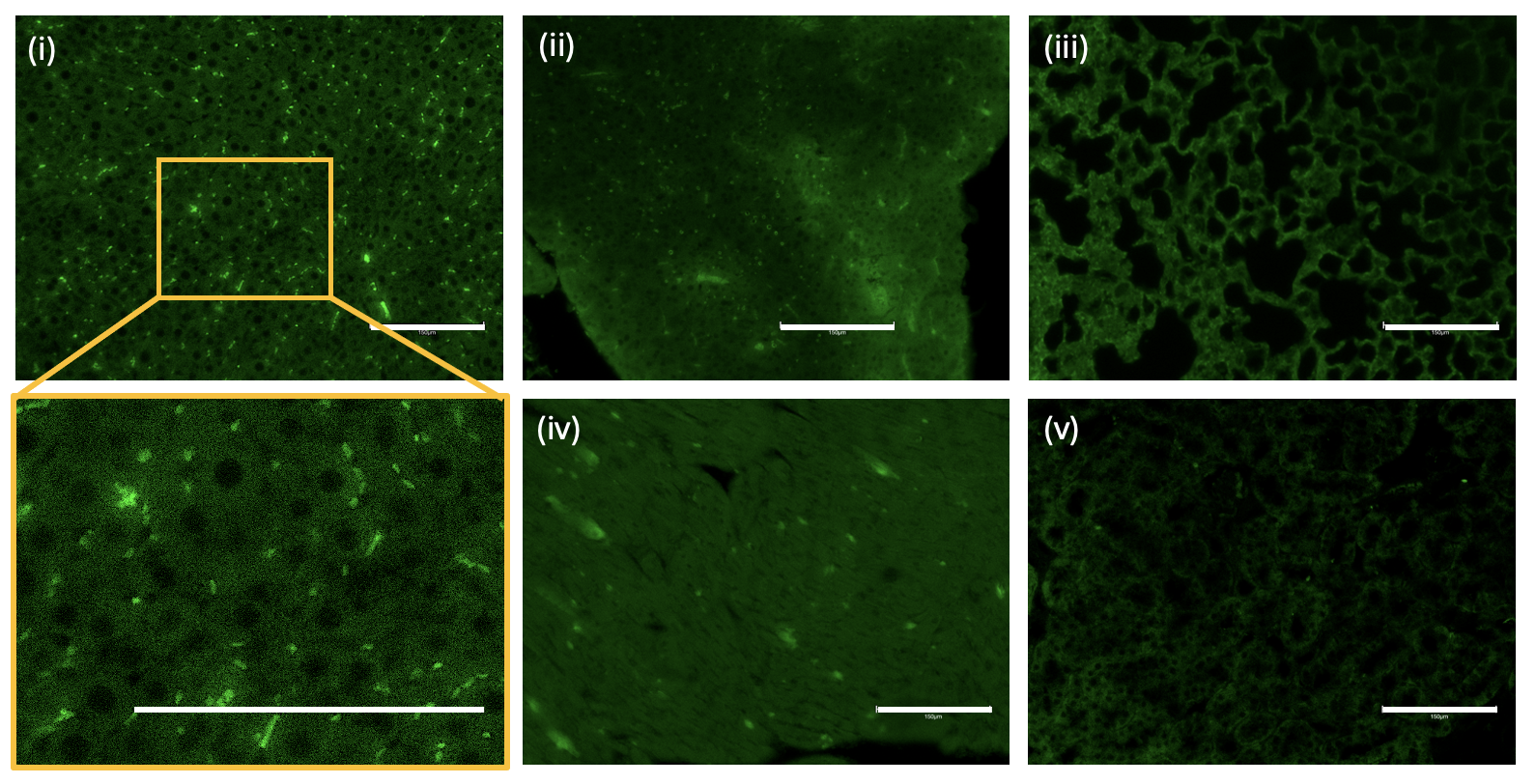


**Fig S9**. Biodistribution of NPs (PC+INT) 8 hours post injection: (a) NP distribution in different organs (i) Liver; (ii) Spleen; (iii) Lung; (iv) Heart; (v) Kidney; scale bar- 150 𝛍m; This preliminary biodistribution study shows that NPs accumulated in the liver in the first 8 h post injection and that there is no non-specific accumulation.


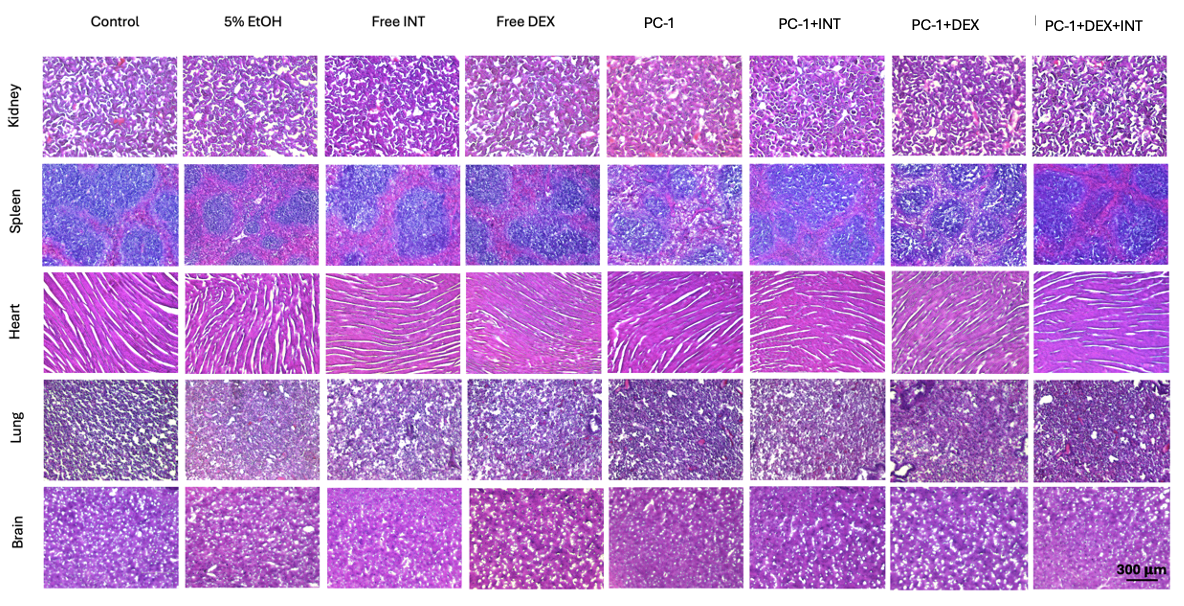


**Fig. S10**. H&E images of brain, lung, heart, spleen and kidneys following different treatments. There is no evidence of inflammation or toxicity in the organs due to the NP treatment.

**Table S1**. Optimization of PC-1+DEX NPs using different solvents; Methanol/DCM=1:9 ratio was proven to be the best combination when considering EE%, size, PDI, and ZP

| **Solvent** | **EE %** | **Loading ug/25 mg of PLGA** | **Particle size before CMC coating (nm)** | **Particle size after CMC coating (nm)** | **After lyophilization** | | |
| --- | --- | --- | --- | --- | --- | --- | --- |
|  |  |  |  |  | Size (nm) | PDI | ZP (mV) |
| **DCM/Acetone 1:1** | 3.88 | 97.09 | 121.95 | 157.40 | 315.88 | 0.38 | -23.23 |
| **Acetone** | 5.79 | 144.85 | 224.45 | 178.23 | 375.00 | 0.41 | -24.93 |
| **Chloroform** | 5.35 | 133.75 | 123.33 | 142.33 | 194.80 | 0.20 | -31.87 |
| **Methanol/DCM 1:9** | 6.24 | 156.23 | 151.05 | 183.95 | 188.35 | 0.17 | -29.00 |

**Table S2**. Statistical significance between experimental groups in IL-6 cytokine release at 6 h timepoint (Figure 5ai); Statistical significance is indicated as *p<0.05, **p<0.01, ***p<0.001, ****p<0.0001.

|  | control | ALD control | Free INT high | Free DEX high | PC-1 NPs high | PC-1+INT high | PC-1+DEX high | PC-1+DEX+INT high |
| --- | --- | --- | --- | --- | --- | --- | --- | --- |
| control |  | ** |  |  |  |  |  |  |
| ALD control | ** |  |  |  |  |  |  |  |
| Free INT low | **** |  | **** | **** | ** | *** | *** | **** |
| Free Dex low |  |  |  |  |  |  |  |  |
| PC-1 NPs low | *** |  | * | * | ** | * | * | * |
| PC-1+INT low | **** |  | *** | *** |  | ** | ** | *** |
| PC-1+DEX low | ** |  | * | * |  |  |  | * |
| PC-1+DEX+INT low | **** |  | *** | *** | ** |  | ** | *** |

|  | control | ALD control | Free INT high | Free DEX high | PC-1 NPs high | PC-1+INT high | PC-1+DEX high | PC-1+DEX+INT high |
| --- | --- | --- | --- | --- | --- | --- | --- | --- |
| control |  | **** | *** |  | * |  |  |  |
| ALD control | **** |  |  |  |  | *** | ** | **** |
| Free INT low |  | **** | ** |  |  |  |  |  |
| Free Dex low |  | *** | ** |  |  |  |  |  |
| PC-1 NPs low |  | *** | ** |  |  |  |  |  |
| PC-1+INT low |  | ** |  |  |  |  |  |  |
| PC-1+DEX low | ** |  |  |  |  | * |  | ** |
| PC-1+DEX+INT low | **** |  |  |  |  | *** | * | **** |

**Table S3**. Statistical significance between experimental groups in IL-6 cytokine release at 24 h timepoint (Figure 5aii); Statistical significance is indicated as *p<0.05, **p<0.01, ***p<0.001, ****p<0.0001.

**Table S4**. Statistical significance between experimental groups in TNF-α cytokine release at 6 h timepoint (Figure 5bi); statistical significance is indicated as *p<0.05, **p<0.01, ***p<0.001, ****p<0.0001.

|  | control | ALD control | Free INT high | Free DEX high | PC-1 NPs high | PC-1+INT high | PC-1+DEX high | PC-1+DEX+ INT high |
| --- | --- | --- | --- | --- | --- | --- | --- | --- |
| control |  | **** | *** |  | **** | *** | ** |  |
| ALD control | **** |  | * | **** |  |  | ** | **** |
| Free INT low | **** |  | *** |  |  |  |  | *** |
| Free Dex low | ** | * |  |  | **** |  |  |  |
| PC-1 NPs low | **** |  | * | **** |  |  | ** | **** |
| PC-1+INT low | **** |  | *** |  |  |  | * | *** |
| PC-1+DEX low | **** |  |  | ** |  |  |  | ** |
| PC-1+DEX+INT low | *** | * |  |  |  | **** |  |  |

**Table S5**. Statistical significance between experimental groups in TNF-α cytokine release at 24 h timepoint (Figure 5bii); statistical significance is indicated as *p<0.05, **p<0.01, ***p<0.001, ****p<0.0001.

|  | control | ALD control | Free INT high | Free DEX high | PC-1 high | PC-1+INT high | PC-1+DEX high | PC-1+DEX+INT high |
| --- | --- | --- | --- | --- | --- | --- | --- | --- |
| control |  | *** |  |  | **** |  |  |  |
| ALD control | *** |  | * | ** |  |  |  | ** |
| Free INT low | * |  |  |  | ** |  |  |  |
| Free Dex low |  |  |  |  | *** |  |  |  |
| PC-1 low | **** |  | *** | **** | ** | ** | ** | *** |
| PC-1+INT low | * |  |  |  |  |  |  |  |
| PC-1+DEX low |  |  | * |  | * |  |  | * |
| PC-1+DEX+INT low |  |  |  |  | ** |  |  |  |

**Table S6**. Statistical significance between experimental groups in IL-1β cytokine release at 6 h timepoint (Figure 5ci); statistical significance is indicated as *p<0.05, **p<0.01, ***p<0.001, ****p<0.0001.

|  | control | ALD control | Free INT high | Free DEX high | PC-1 NPs high | PC-1+INT high | PC-1+DEX high | PC-1+DEX+INT high |
| --- | --- | --- | --- | --- | --- | --- | --- | --- |
| control |  | **** | **** | **** | **** | **** | **** | **** |
| ALD control | **** |  |  |  |  |  | *** | * |
| Free INT low | **** | * |  |  |  |  |  |  |
| Free Dex low | *** | **** | ** | *** |  | **** |  |  |
| PC-1 NPs low | **** |  |  |  |  |  |  |  |
| PC-1+INT low | **** |  |  |  |  |  | * |  |
| PC-1+DEX low | **** | **** | ** | *** | **** | **** |  |  |
| PC-1+DEX+INT low | **** | * |  |  |  |  |  |  |

**Table S7**. Statistical significance between experimental groups in IL-1β cytokine release at 24 h timepoint (Figure 5cii); Statistical significance is indicated as *p<0.05, **p<0.01, ***p<0.001, ****p<0.0001.

|  | control | ALD control | Free INT high | Free DEX high | PC-1 NPs high | PC-1+INT high | PC-1+DEX high | PC-1+DEX+INT high |
| --- | --- | --- | --- | --- | --- | --- | --- | --- |
| control |  | **** | **** |  | **** | **** | ** |  |
| ALD control | **** |  | *** |  |  |  | * | **** |
| Free INT low | **** |  |  | * | ** | *** |  | ** |
| Free Dex low | * | ** | *** |  | **** | **** |  |  |
| PC-1 NPs low | **** |  |  | **** |  |  | ** | **** |
| PC-1+INT low | **** |  |  | **** |  |  | **** |  |
| PC-1+DEX low | ** | ** | ** |  | **** | **** |  |  |
| PC-1+DEX+INT low | **** |  |  |  | *** | *** |  | * |

**Table S8**. Statistical significance between experimental groups in cAMP activity at 6 h timepoint (Figure 5di); Statistical significance was indicated as *p<0.05, **p<0.01, ***p<0.001, ****p<0.0001.

|  | control | ALD control | Free INT high | Free DEX high | PC-1 NPs high | PC-1+INT high | PC-1+DEX high | PC-1+DEX+INT high |
| --- | --- | --- | --- | --- | --- | --- | --- | --- |
| control |  | *** |  |  |  |  | * |  |
| ALD control | *** |  |  |  |  |  |  | * |
| Free INT low | ** |  |  |  |  |  |  |  |
| Free Dex low | ** |  |  |  |  |  |  |  |
| PC-1 NPs low | ** |  |  |  |  |  |  |  |
| PC-1+INT low | ** |  |  |  |  |  |  |  |
| PC-1+DEX low |  |  |  |  |  |  |  |  |
| PC-1+DEX+INT low |  |  |  |  |  |  |  |  |

**Table S9**. Statistical significance between experimental groups in cAMP activity at 6 h timepoint (Figure 5dii); statistical significance is indicated as *p<0.05, **p<0.01, ***p<0.001, ****p<0.0001.

|  | control | ALD control | Free INT high | Free DEX high | PC-1 NPs high | PC-1+INT high | PC-1+DEX high | PC-1+DEX+INT high |
| --- | --- | --- | --- | --- | --- | --- | --- | --- |
| control |  | **** | **** | **** | **** | **** | **** | **** |
| ALD control | *** |  | **** | *** |  |  |  | **** |
| Free INT low | **** |  | **** |  | * | * |  | **** |
| Free Dex low | **** |  |  | *** |  |  |  | *** |
| PC-1 NPs low | **** |  | **** |  |  |  |  | **** |
| PC-1+INT low | **** |  | **** |  |  |  |  | **** |
| PC-1+DEX low | **** |  | ** | * |  |  |  | **** |
| PC-1+DEX+INT low | **** |  |  |  |  |  |  | **** |
